# Human JAK1 gain of function causes dysregulated myelopoeisis and severe allergic inflammation

**DOI:** 10.1172/jci.insight.150849

**Published:** 2022-12-22

**Authors:** Catherine M. Biggs, Anna Cordeiro-Santanach, Sergey V. Prykhozhij, Adam P. Deveau, Yi Lin, Kate L. Del Bel, Felix Orben, Robert J. Ragotte, Aabida Saferali, Sara Mostafavi, Louie Dinh, Darlene Dai, Katja G. Weinacht, Kerry Dobbs, Lisa Ott de Bruin, Mehul Sharma, Kevin Tsai, John J. Priatel, Richard A. Schreiber, Jacob Rozmus, Martin C.K. Hosking, Kevin E. Shopsowitz, Margaret L. McKinnon, Suzanne Vercauteren, Michael Seear, Luigi D. Notarangelo, Francis C. Lynn, Jason N. Berman, Stuart E. Turvey

**Affiliations:** 1 Department of Pediatrics, University of British Columbia, Vancouver, British Columbia, Canada.; 2 BC Children’s Hospital, Vancouver, British Columbia, Canada.; 3 AGADA Biosciences Inc., Halifax, Nova Scotia, Canada.; 4 CHEO Research Institute, University of Ottawa, Ottawa, Ontario, Canada.; 5 Department of Internal Medicine, Dalhousie University, Halifax, Nova Scotia, Canada.; 6 Channing Division of Network Medicine, Brigham and Women’s Hospital, Harvard Medical School, Boston, Massachusetts, USA.; 7 Department of Medical Genetics and; 8 Department of Statistics, University of British Columbia, Vancouver, British Columbia, Canada.; 9 Division of Stem Cell Transplantation and Regenerative Medicine, Department of Pediatrics, Stanford School of Medicine, Stanford, California, USA.; 10 Laboratory of Clinical Immunology and Microbiology, National Institute of Allergy and Infectious Diseases (NIAID), NIH, Bethesda, Maryland, USA.; 11 Division of Immunology, Boston Children’s Hospital, Harvard Medical School, Boston, Massachusetts, USA.; 12 Department of Pathology and Laboratory Medicine and; 13 Department of Surgery, University of British Columbia, Vancouver, British Columbia, Canada.; 14 Departments of Pediatrics and Cellular and Molecular Medicine, University of Ottawa, Ottawa, Ontario, Canada.

**Keywords:** Hematology, Immunology, Allergy, Bone marrow differentiation

## Abstract

Primary atopic disorders are a group of inborn errors of immunity that skew the immune system toward severe allergic disease. Defining the biology underlying these extreme monogenic phenotypes reveals shared mechanisms underlying common polygenic allergic disease and identifies potential drug targets. Germline gain-of-function (GOF) variants in *JAK1* are a cause of severe atopy and eosinophilia. Modeling the *JAK1*^GOF^ (p.A634D) variant in both zebrafish and human induced pluripotent stem cells (iPSCs) revealed enhanced myelopoiesis. RNA-Seq of *JAK1*^GOF^ human whole blood, iPSCs, and transgenic zebrafish revealed a shared core set of dysregulated genes involved in IL-4, IL-13, and IFN signaling. Immunophenotypic and transcriptomic analysis of patients carrying a *JAK1*^GOF^ variant revealed marked Th cell skewing. Moreover, long-term ruxolitinib treatment of 2 children carrying the *JAK1*^GOF^ (p.A634D) variant remarkably improved their growth, eosinophilia, and clinical features of allergic inflammation. This work highlights the role of JAK1 signaling in atopic immune dysregulation and the clinical impact of JAK1/2 inhibition in treating eosinophilic and allergic disease.

## Graphical abstract

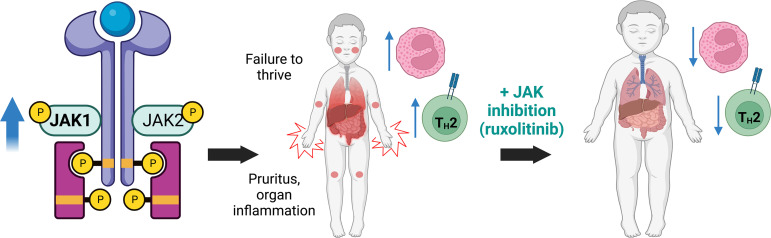

## Introduction

Asthma and allergies cause immense worldwide health and economic burdens (1). Understanding the cellular and molecular mechanisms driving allergic inflammation is a critical step in identifying new treatment and preventive measures. Studying rare human patients with monogenic inborn errors of immunity (IEIs) associated with eosinophilia and allergic inflammation provides unique insights into the molecular mechanisms underlying atopy and may identify new therapeutic targets.

In 2017, an IEI was discovered in a family with severe atopic dermatitis, asthma, food allergy, failure to thrive, autoimmune thyroiditis, and markedly elevated peripheral blood eosinophil counts (2). Whole exome sequencing revealed a heterozygous gain-of-function (GOF) variant in the regulatory pseudokinase domain of *JAK1* (*JAK1*^GOF^) in affected family members (c.1901C>A, p.A634D). JAK1 belongs to the JAK family of protein kinases, which are employed by multiple cytokine and growth factor receptors involved in cellular immunity, hematopoiesis, and growth (3). Further confirming this powerful genotype-phenotype relationship, an unrelated patient with eosinophilia and severe allergic inflammation was also found to be heterozygous for a missense *JAK1*^GOF^ variant located in the pseudokinase domain (4). Similarly, 2 different mouse strains with *JAK1*^GOF^ variants induced by N-ethyl-N-nitrosourea (ENU) mutagenesis (i.e., *Spade* R878H and S645P) also developed allergic manifestations including atopic dermatitis, elevated IgE and a Th2-biased immune system (5, 6)

Eosinophils are myeloid-derived cells involved in the regulation of local inflammatory responses and are key effectors in allergic disease (7–10). Despite their role in immunity, defining the signaling pathways responsible for eosinopoiesis remains elusive. Recent studies have identified the importance of IL-33 in early eosinophil commitment; however, the factors driving IL-33 receptor expression in myeloid precursors are unknown (11, 12). IL-5 is an established regulator of eosinophil differentiation and proliferation (13). IL-3 and GM-CSF, whose receptors share a common β chain with IL-5, also contribute to both eosinophil production and survival (9).

Currently, there is no clear mechanistic biological link between JAK1 and hypereosinophilia. JAK1 has no known role in regulating IL-33 receptor expression, and GM-CSF, IL-3, and IL-5 are thought to primarily signal through JAK2 (3). Previous studies focused on the importance of JAK1 in type 1 IFN responses against viruses. Although dysregulated antiviral responses are implicated in allergic skewing in early life, the signals driving this paradox are unclear (14). Combining insights from studies on human whole blood, induced pluripotent stem cells (iPSC) and zebrafish carrying the *JAK1*^GOF^ variant, we sought to investigate how enhanced JAK1 signaling promotes allergic inflammation. Despite the established role for JAK1 in IL-6 and IFN signaling, we determined that heightened JAK1 signaling drives a Th2 phenotype, explaining the clinical findings of severe allergic disease in *JAK1*^GOF^ patients. In addition, long-term data on Jakinib use in children are sparse, and studies in myeloproliferative neoplasms have raised concerns surrounding infectious risks, most notably to viruses (15). Here, we describe the outcomes of *JAK1*^GOF^ patients treated with ruxolitinib for 6 years, contributing important safety and clinical data for pediatric patients treated with Jakinibs. Together these findings have important implications for our understanding of the pathogenesis and potential therapeutic targets for burdensome human allergic immune dysregulation, particularly in light of the growing number of Jakinibs now approved for clinical use (16).

## Results

### Allergic immune dysregulation and hematologic abnormalities in patients with JAK1^GOF^ and long-term sustained response to ruxolitinib.

**Figure 1:**
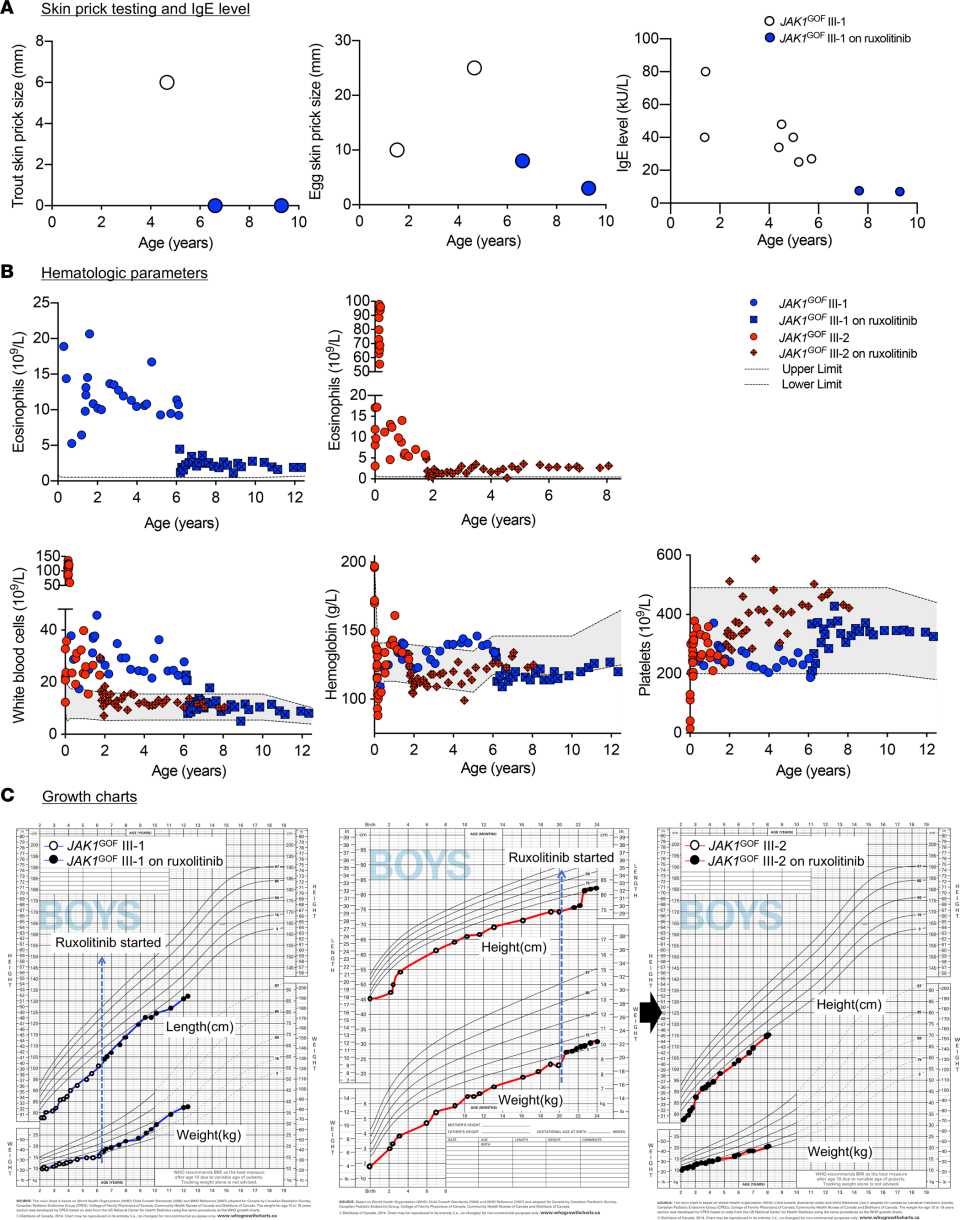
Sustained clinical improvement of *JAK1^GOF^* patients on ruxolitinib. (**A**) Decline in both IgE level and skin prick test sizes in patient III-1 following ruxolitinib treatment. (**B**) Affected patients showed severe elevations in total WBC count, followed by normalization with ruxolitinib. Hemoglobin levels were normal/elevated prior to ruxolitinib, after which they remained on the lower limit of normal/mildly low. Platelet counts remained within the normal range; however, they were higher in number on ruxolitinib treatment. Ruxolitinib therapy dramatically improved eosinophil counts; however, they remained above the normal upper limit (0.5 × 10^9^/L). (**C**) Growth charts of patients III-I and III-2 carrying the *JAK1^GOF^* variant before and after treatment with ruxolitinib.

We carefully evaluated for allergic inflammation in 2 *JAK1*^GOF^-affected children (designated III-1 and III-2 in the family pedigree) (Supplemental Figure 1; supplemental material available online with this article; https://doi.org/10.1172/jci.insight.150849DS1) and their affected mother (II-2). As previously reported, clinical evaluation revealed that both children had suffered with severe, treatment-resistant atopic dermatitis and severe asthma prior to beginning targeted therapy with ruxolitinib (2). Ruxolitinib treatment initiated at ages 6 years (III-1) and 20 months (III-2) rapidly improved pruritus and atopic dermatitis symptoms, so that topical corticosteroids were discontinued. While respiratory symptoms also improved with ruxolitinib, both patients remain on inhaled fluticasone/salmeterol. Pulmonary function testing on patient III-1 continues to show obstructive airway disease with significant bronchodilator response; there is also a component of nonreversible obstruction, attributed to chronic lung disease. Patient III-2 experienced severe parainfluenza infection at age 8 months; since initiating ruxolitinib, he has not had any significant respiratory exacerbations and has normal spirometry. Patient III-1 has a history of fish allergy diagnosed based on history of anaphylaxis following fish exposure and positive skin prick testing at 2 years of age; recent reevaluation shows negative skin testing and normalization of total IgE (Figure 1A). Patient III-2, who began ruxolitinib at a much younger age than patient III-1, has not developed any specific allergies. Patient III-1 has also suffered from episodes of acute idiopathic urticaria. Periodic breakthrough of pruritus and loss of appetite/feeding intolerance have been successfully managed with modest uptitration of ruxolitinib dosing; currently, the patients are well controlled on a ruxolitinib dose of 1.5–2.5mg/kg/day divided twice daily. Similar to III-1 and III-2, their mother (patient II-2) suffers from severe allergic inflammation, including eosinophilia, atopic dermatitis, asthma, allergic rhinitis, and food and drug allergy. Treatment with a selective JAK1 inhibitor upadacitinib at a dose of 15 mg/day improved the patient’s pruritis and dermatitis (although to a lesser extent than the benefits her sons experienced with ruxolitinib); however, it was associated with side effects of rosacea and weight gain.

Prior to initiating ruxolitinib treatment, affected patients demonstrated elevated hematopoietic lineages, except for platelets, which were severely decreased at birth in patient III-2 and were subsequently generally normal in number for both patients III-1 and III-2 (Figure 1B). Interestingly, platelet numbers appear to trend upward upon treatment with ruxolitinib, in contrast to WBC and hemoglobin levels, which declined. Affected patients demonstrated monocytosis, intermittent neutrophilia and lymphocytosis, and severe eosinophilia preruxolitinib (Figure 1B and Supplemental Figure 2) (2). Although eosinophil counts dramatically decreased upon treatment with ruxolitinib, they remain moderately elevated, with dose-dependent anemia limiting further increases in ruxolitinib dosing. Decreased hemoglobin was an anticipated side effect, as ruxolitinib will inhibit erythropoietin signaling through JAK2.

Growth parameters are an effective metric for overall health in the pediatric population. Growth failure began in utero in *JAK1*^GOF^ patients, followed in the postnatal period by short stature and poor weight gain. Prior to the discovery of the *JAK1*^GOF^ variant, patient III-1 underwent clinical evaluation for growth failure, and the growth hormone (GH) signaling axis was assessed by measuring serum levels of insulin-like growth factor (IGF-1), which is produced by the liver in response to GH. IGF-1 levels were undetectable, and on repeat analysis, they were severely decreased (undetectable and 33 μg/L; age-specific reference interval, 62–361 μg/L). Ruxolitinib treatment initiation in patients III-1 and III-2 was followed by marked and sustained improvement in both height and weight accrual velocity (Figure 1C) (2). Reanalysis of the GH axis in patient III-1 following ruxolitinib treatment, however, demonstrated normalization of IGF-1 (63–138 μg/L); this was unanticipated since GH signals primarily through JAK2 and was predicted to be inhibited by ruxolitinib, which targets both JAK1 and JAK2 signaling (17). Both patients were also diagnosed with hypothyroidism, eosinophilic gastrointestinal disease (EGID), and possible liver fibrosis. After starting ruxolitinib, they had rapid reversal of gastrointestinal symptoms (vomiting and feeding intolerance), as well as improvement in liver enzymes and fibrosis markers. Additional clinical features seen in these patients include cardiac (patent foramen ovale in III-1 and atrial septal defect in III-2), vascular (abnormal course of inferior vena cava in III-1 and III-2), and structural brain anomalies (chiari malformation in III-1 and high riding jugular bulb with conductive hearing loss in III-2).

### JAK1^GOF^ drives myelopoiesis in humans.

**Figure 2:**
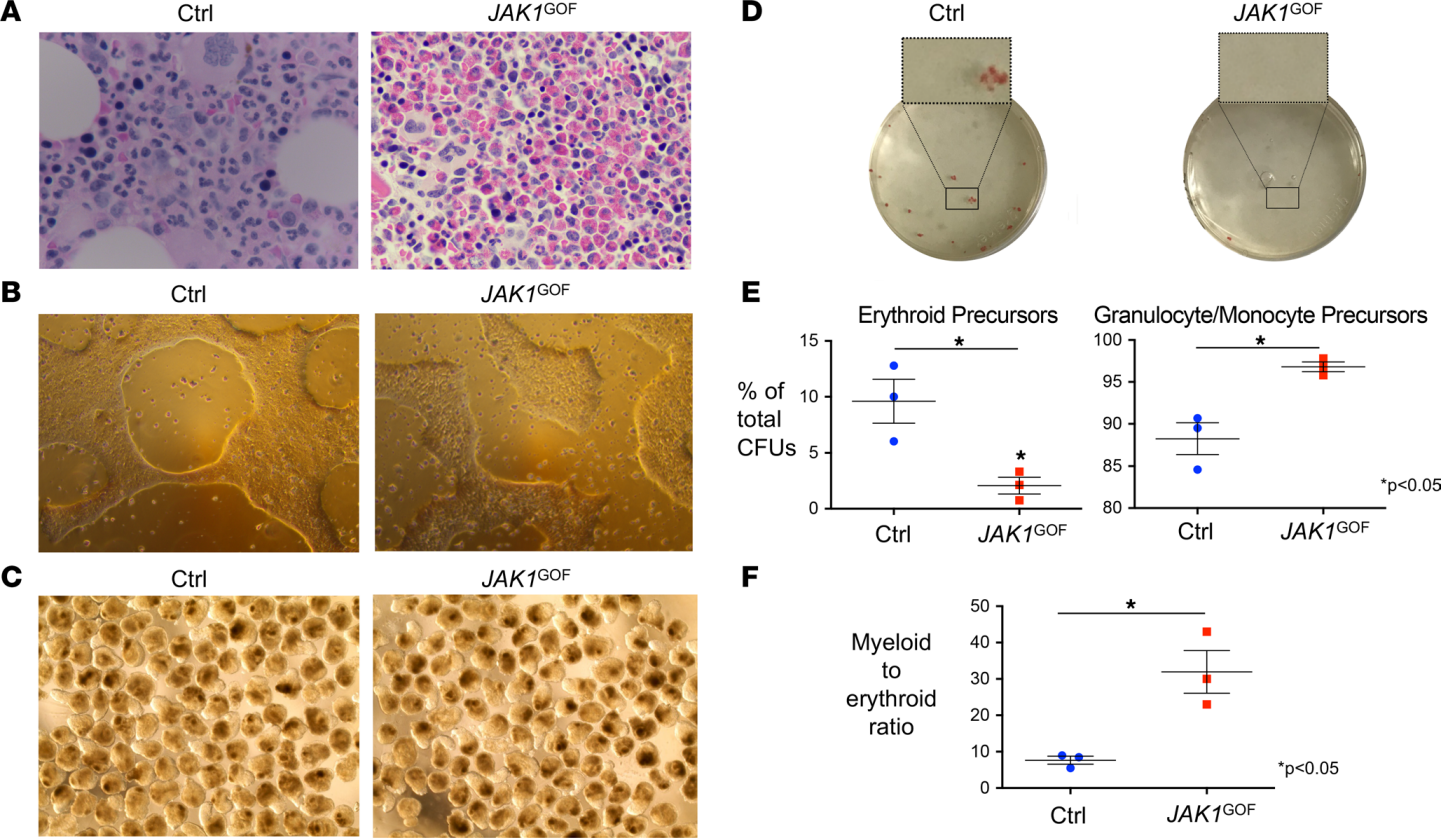
*JAK1*^GOF^ BM and iPSC reveal skewing toward myelopoiesis. (**A**) BM biopsy slide obtained from *JAK1*^GOF^ patient demonstrates an increase in the eosinophil lineage compared with healthy control BM biopsy (Ctrl). Images are at 50× magnification. (**B**) Representative photographs of *JAK1*^GOF^ and XY control (Ctrl) iPSCs, demonstrating normal morphology for iPSCs grown in feeder-free culture, including tightly packed iPSCs with well-defined edges. (**C**) Embryoid body (EB) differentiation of *JAK1*^GOF^ and Ctrl iPSCs. Images obtained on day 10 of EB differentiation. (**D**) Representative methylcellulose plates with hematopoietic precursor CFUs, demonstrating increased proportion of hemoglobinized erythroid precursor CFUs in Ctrl compared with *JAK1*^GOF^. Experiments represented in **B**–**D** were performed 3 times. (**E**) Decreased proportion of erythroid (CFU-E, BFU-E) CFUs in *JAK1*^GOF^ compared with Ctrl, with an increased proportion of granulocyte/monocyte precursors (CFU-G, CFU-M, CFU-GM). Average percentage of total CFUs (data presented as mean ± SEM) was compared using a 2-tailed unpaired *t* test with Welch’s correction. (**F**) Increased myeloid/erythroid ratio (data presented as mean ± SEM) in *JAK1*^GOF^ compared with Ctrl using a 2-tailed unpaired *t* test with Welch’s correction.

Given the striking eosinophilia seen in affected patients, we evaluated the impact of *JAK1*^GOF^ on hematopoiesis. BM studies from the 2 children carrying the germline *JAK1*^GOF^ variant (designated III-1 and III-2) revealed increased eosinophils and eosinophilic precursors; formal BM enumeration from 1 affected patient demonstrated an elevated myeloid/erythroid ratio of 9:1 (age-reference average 3.5:1) (18) with 69% of BM cells belonging to the eosinophil lineage (age-reference range, 0%–6%) (19) (Figure 2A). Directed myeloid differentiation performed on *JAK1*^GOF^ and XY control iPSCs (Figure 2, B and C) further confirmed this skewing toward myelopoiesis, with an increased proportion of myeloid colonies and an increased myeloid/erythroid colony ratio in *JAK1*^GOF^ samples compared with controls (Figure 2, D–F).

### Skewed Th subsets in patients carrying the JAK1^GOF^ variant responsive to Jakinib therapy.

**Figure 3:**
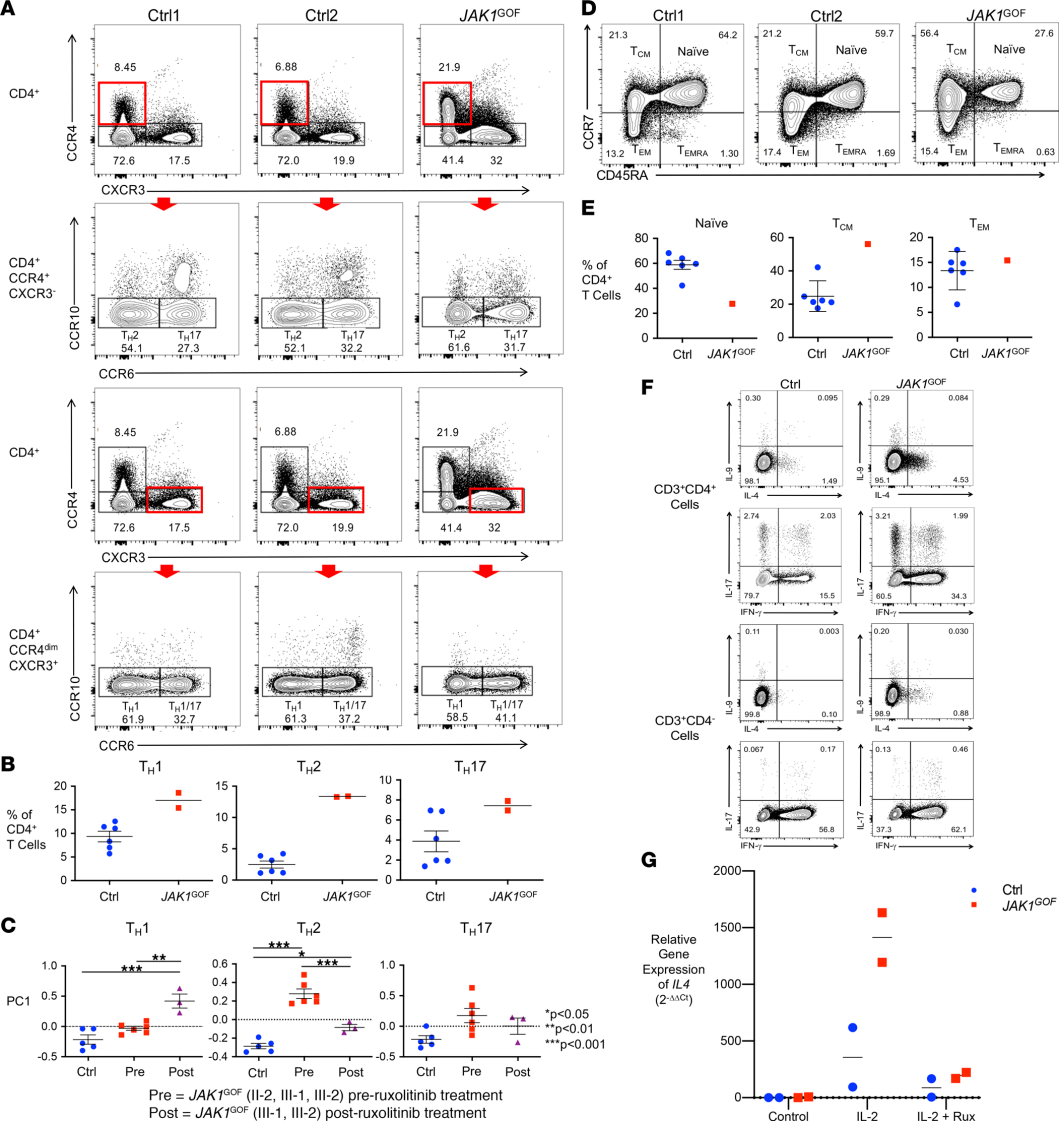
Enhanced Th2 phenotype and T cell activation in *JAK1*^GOF^. (**A**) Representative flow cytometry plots of healthy controls (Ctrl) and *JAK1*^GOF^ patient (II-2) Th2, Th17, Th1, and Th1/17 cell subsets as a proportion of live CD3^+^CD4^+^ T cells, identified as CCR4^+^CXCR3^–^CCR10^–^CCR6^–^, CCR4^+^CXCR3^–^CCR10^–^CCR6^+^, CCR4^–^CXCR3^+^CCR10^–^CCR6^–^, and CCR4^–^CXCR3^+^CCR10^–^CCR6^+^, respectively. The red rectangles highlight the cell population gated on for the subsequent plot identified by the red arrow. This experiment was performed twice with patient samples. (**B**) Average frequency of Th subsets (data presented as mean ± SEM). Subset frequency calculated by multiplying the proportion of each subset by the sample’s number of CD4^+^ T cells. (**C**) PCA of Th subset gene expression from whole blood RNA-Seq data. First principal component (PC) values (data presented as mean ± SEM) were compared between groups using 1-way ANOVA with Bonferri multiple comparisons correction. (**D**) Decreased proportion of naive CD4^+^ *JAK1*^GOF^ T cells. Representative flow cytometry of and (**E**) proportions of naive, effector memory (T_EM_), effector memory reexpressing CD45RA (T_EMRA_), and central memory (T_CM_) CD4^+^ T cells. This experiment was performed once with patient cells. (**F**) Increased frequency of IL-4–, IFN-γ–, and IL-17–secreting *JAK1*^GOF^ CD4^+^ T cells and IL-4–, IL-9–, and IL-17–secreting *JAK1*^GOF^ CD8^+^ T cells (identified as CD3^+^CD4^–^ T cells) compared with control. (**G**) Increased *IL4* gene expression in expanded and activated *JAK1*^GOF^ T cells. Measured using qPCR after 4 hours of stimulation with DMSO control, IL-2, and IL-2 plus ruxolitinib (1 mM). Relative gene expression Ctrl and *JAK1*^GOF^ were calculated using the Livak method (2^–ΔΔCt^).

We then evaluated the effect of *JAK1*^GOF^ on lymphocytes. Multiple aspects of the *JAK1*^GOF^ clinical phenotype suggest T cell dysregulation, including the atopy and autoimmune thyroid disease seen in all affected family members. To determine the impact of enhanced JAK1 signaling on T cell differentiation, we analyzed the proportion of Th cell subsets in peripheral blood mononuclear cells (PBMCs) isolated from a *JAK1*^GOF^ patient (designated II-2 in the family pedigree; Supplemental Figure 1) who had not received Jakinib treatment, in comparison with healthy controls (HCs). We identified increased Th2, Th17, and Th1 subsets in the *JAK1*^GOF^ patient, with the most dramatic effect observed in the Th2 compartment (Figure 3, A and B). To further analyze the effect of enhanced JAK1 signaling on Th differentiation using a different experimental approach, we leveraged the whole blood RNA-Seq data obtained from HCs and *JAK1*^GOF^ patients (Figure 3C). In order to evaluate Th cells within the cellular heterogeneity of whole blood expression data, we focused our analysis on genes that have been previously identified in the literature as specific for various Th populations (20). Principal component analysis (PCA) was performed comparing established gene expression signatures of Th1, Th2, and Th17 subsets between patients and HCs (20). Recapitulating the flow cytometry–based T cell phenotyping, we detected a significant increase in the Th2 gene signature expression in the presence of the *JAK1*^GOF^ variant compared with HCs (Figure 3C). Importantly, ruxolitinib treatment significantly decreased the in vivo Th2 gene signature of *JAK1*^GOF^ whole blood, while the Th1 gene signature increased.

To determine the impact of enhanced JAK1 signaling on T cell homeostasis and function, we performed additional analyses on T cells from a *JAK1*^GOF^ patient (II-2) who had not received Jakinib treatment (Figure 3, D–G). First, we analyzed the proportions of naive and T cell memory populations compared with age-matched HCs (Figure 3, D and E). Consistent with the finding of increased Th subsets in *JAK1*^GOF^ T cells identified by transcriptomics, a decreased proportion of naive CD4^+^ T cells, along with concomitant increased representation of effector and central memory T cells, was observed among *JAK1*^GOF^ PBMCs relative to HCs. Assessing function using expanded T cells, following 4 hours of PMA/ionomycin stimulation, the *JAK1*^GOF^ patient had an increased proportion of IL-4–, IFN-γ–, and IL-17–producing CD4^+^ T cells, with the largest difference seen in the IL-4^+^ population (Figure 3F). To evaluate CD8^+^ T cell cytokine expression, gating on CD3^+^CD4^–^ T cells revealed increased IL-4 and IL-17 and similar IFN-γ expression when comparing *JAK1*^GOF^ cells to HC. It must be noted that this experiment was only performed once due to limitations in patient sample availability as the patient began therapy with a JAK1 inhibitor (upadacitinib). Following treatment for 10 months with upadacitinib, repeat analysis showed a modest decrease in the proportion of IL-4–, IL-17–, and IFN-γ–producing CD4^+^ T cells in the affected patient compared with pretreatment values, with a greater proportional decline observed in IL-4 and IL-17; however, the frequency of IL-4 and IFN-γ secreting CD4^+^ T cells was still elevated compared with control (Supplemental Figure 3). Validating the cytokine production data of samples taken pretreatment, *IL4* gene expression was also assessed by quantitative PCR (qPCR) following 4 hours of treatment with IL-2, IL-2 with ruxolitinib, or a vehicle control (DMSO). In comparison with the HC T cells, *JAK1*^GOF^ patient T cells showed 3-fold higher expression of *IL4* in response to stimulation with IL-2, with ruxolitinib decreasing this response to baseline levels in both HC and *JAK1*^GOF^ T cells (Figure 3G).

### Abnormal transcriptome and cytokine receptor profile in human JAK1^GOF^.

**Figure 4:**
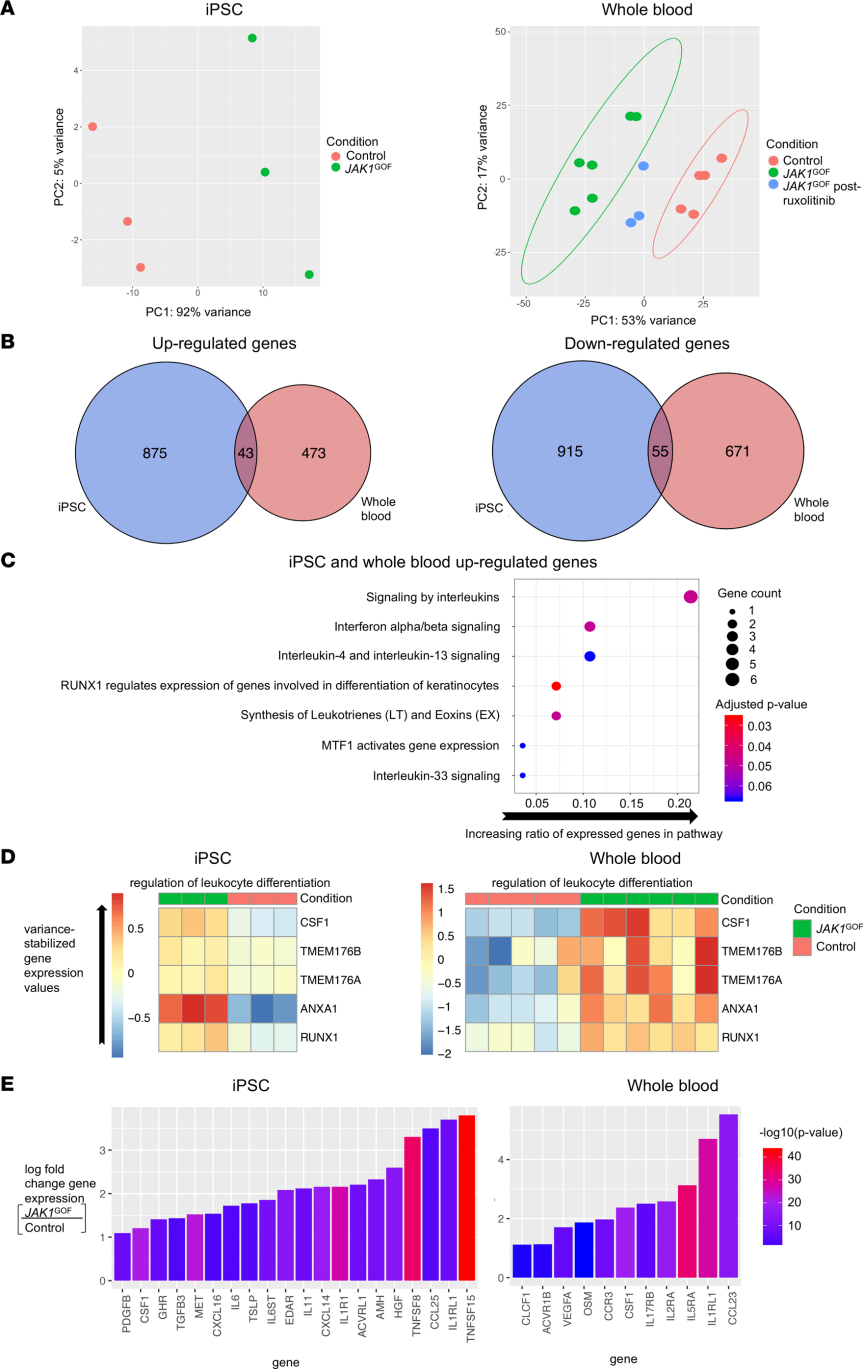
Abnormal gene expression and enhanced cytokine signaling in human *JAK1*^GOF^. (**A**) PCA of iPSC and whole blood showing clustering of *JAK1*^GOF^ compared with controls, with *JAK1*^GOF^ samples from patients following treatment with ruxolitinib shifting closer to controls. Ellipses demonstrating independent clustering are shown for groups containing more than 3 samples. (**B**) Analysis of differentially expressed genes in human iPSC and whole blood carrying the *JAK1*^GOF^ demonstrated 43 upregulated and 55 downregulated genes in both iPSC and whole blood compared with controls. (**C**) Curated list of significantly enriched pathways performed by Reactome Pathway analysis in the 43 upregulated genes found in both human *JAK1*^GOF^ iPSC and whole blood samples. The *y* axis indicates Reactome Pathway, and the *x* axis corresponds to ratio of upregulated genes to the number of genes in each pathway, and the size of each dot corresponds to number of genes identified in samples. Color corresponds to the adjusted *P* value, with red being more significant. (**D**) Heatmap of variance-stabilized gene expression values from ontology term groups that were enriched in the shared upregulated genes in human *JAK1*^GOF^ iPSC and whole blood samples. (**E**) Waterfall plots of cytokine and cytokine receptors upregulated in human *JAK1*^GOF^ iPSC and whole blood samples. The *y* axis corresponds to the log fold change of *JAK1*^GOF^ compared with controls, and the *x* axis corresponds to gene name; colors indicate –log(*P* value), with red being more significant. Whole blood RNA-Seq data obtained from 5 healthy controls, 3 *JAK1*^GOF^ patients (2 samples each from II-2, III-1, III-2) obtained at different time points before treatment, and 2 *JAK1*^GOF^ patients (III-1, III-2) after treatment (1 sample from III-1 and 2 samples from III-2 obtained at different time points). iPSC RNA-Seq data were obtained from iPSC-derived EBs on day 7 of differentiation (3 samples each from *JAK1*^GOF^ and Ctrl, with 50 EBs collected per sample).

We used transcriptome analysis to generate a global overview of the impact of enhanced JAK1 signaling on hematopoiesis in iPSC-derived embryoid bodies (EBs) undergoing myeloid differentiation (*JAK1*^GOF^ iPSC) and patient-derived whole blood. *JAK1*^GOF^ iPSCs demonstrated a unique gene expression profile based on PCA (Figure 4A). Similarly, patients carrying the *JAK1*^GOF^ variant also demonstrated a unique gene expression signature, and after a minimum of 6 weeks of orally administered systemic therapy with ruxolitinib, their pattern of differential gene expression shifted toward that seen in HCs (Figure 4A). Focusing on alterations specific to the hematopoietic lineage, we evaluated genes that are differentially expressed in both iPSCs and whole blood (Figure 4B). Of the 43 genes that were upregulated in both human *JAK1*^GOF^ iPSCs and whole blood, Reactome Pathway and Gene Ontology (GO) term group analysis revealed enrichment in cytokine signaling and leukocyte differentiation transcriptional programs (Figure 4, C and D, and Supplemental Tables 1 and 2). In addition to IFN and IL pathways known to signal through the JAK/STAT pathway, Reactome analysis revealed enrichment of leukotriene/eoxin and IL-33 signaling, suggesting unexpected mechanisms by which enhanced JAK1 signaling may drive allergic inflammation and eosinophilia.

We then filtered the differential gene expression results for cytokines and cytokine receptors upregulated in either *JAK1*^GOF^ iPSC or whole blood. Twenty cytokines/cytokine receptors were upregulated in *JAK1*^GOF^ iPSCs, and 11 were upregulated in whole blood, the majority of which have been previously implicated in allergic inflammation (Figure 4E and Supplemental Tables 3 and 4). The *TNFSF15* gene encoding TL1A showed the highest upregulation in *JAK1*^GOF^ iPSCs, while the *CCL23* gene showed the highest upregulation in whole blood from *JAK1*^GOF^ patients. The gene encoding the IL-33R (*IL1RL1*) was notable for being highly upregulated in both cell populations (Figure 4E).

### Successful modeling of JAK1^GOF^ using transparent zebrafish.

**Figure 5:**
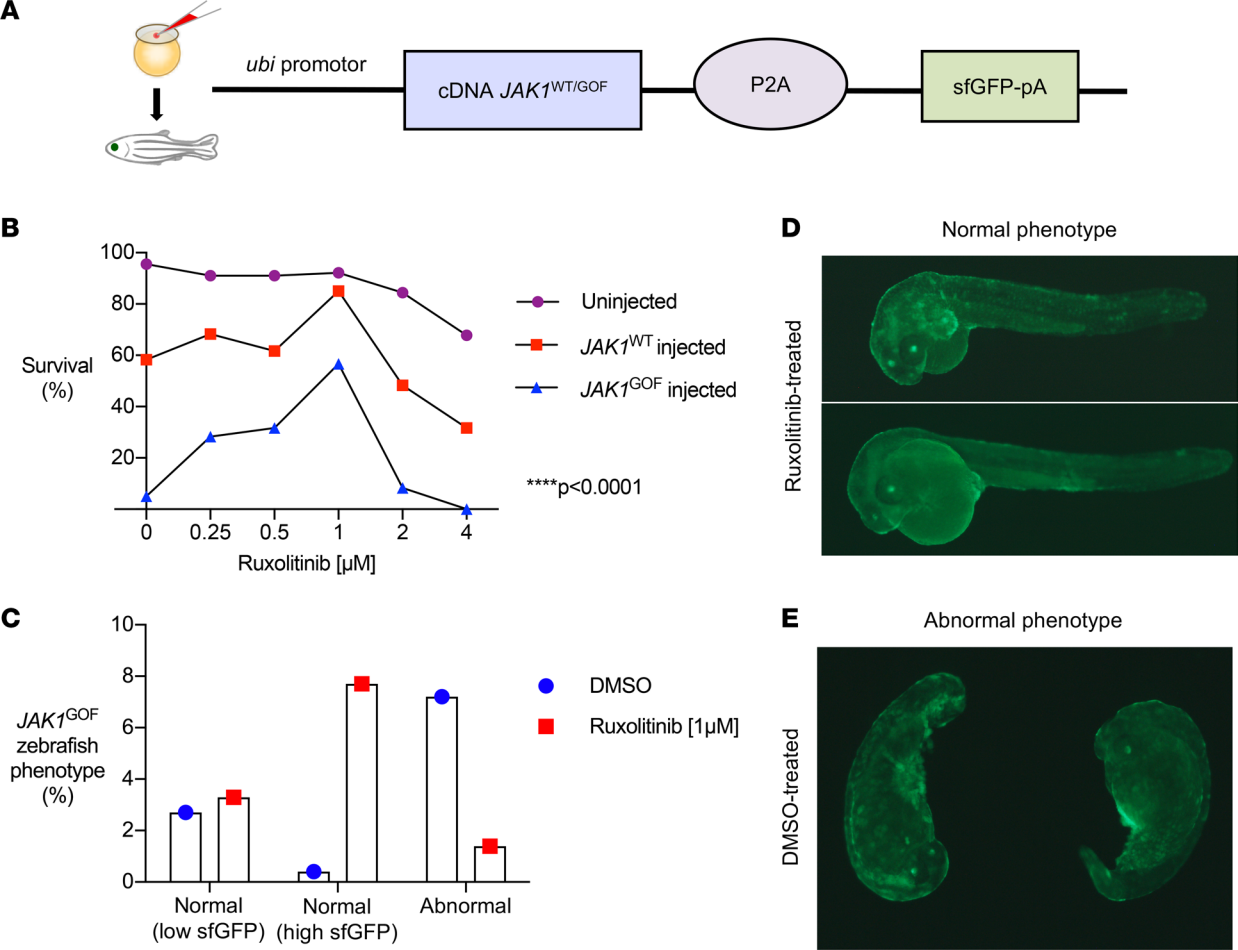
*JAK1* transgenic zebrafish generation. (**A**) We generated 2 zebrafish lines containing human cDNA for JAK1-WT (*JAK1*^WT^) and JAK1-A634D (*JAK1*^GOF^). Ubi is a ubiquitous promoter driving expression of JAK1 and the green fluorescent protein (GFP). Constructs were microinjected into 1-cell stage zebrafish embryos. GFP^+^ fish were selected. (**B**) Different doses of ruxolitinib were tested to determine the optimal dose for the survival for both transgenic fish lines. Ruxolitinib (1 μM) was selected based on higher survival levels for all 3 transgenic lines. Survival was compared between groups (*JAK1*^WT^ and *JAK1*^GOF^ transgenic and uninjected zebrafish) (*P* < 0.0001) and was also compared across varying doses of ruxolitinib in *JAK1*^GOF^ transgenic zebrafish (*P* < 0.0001) using 2-way repeated-measures ANOVA. (**C**) F1 embryos from *JAK1*^GOF^ founders with transgene expression exhibited abnormal development, which was rescued upon exposure to ruxolitinib, revealing a conserved functional developmental impact of *JAK1*^GOF^ in the zebrafish. The remaining percentages correspond to GFP-negative zebrafish. (**D** and **E**) Representative pictures of 30 hpf *JAK1*^GOF^-injected zebrafish, with normal and abnormal development.

To further understand the mechanisms underlying p.A634D JAK1^GOF^–mediated allergic inflammation, we evaluated the effects of enhanced JAK1 signaling on hematopoiesis using zebrafish (*Danio rerio*) — a powerful model of human blood disease (21–25). We generated transgenic lines carrying human *JAK1*^WT^ or *JAK1*^GOF^ genes fused to sfGFP in a *casper* (*mitfa*^w2/w2^; *mpv17*^a9/a9^) zebrafish strain; a transparent (except for eyes) zebrafish mutant that facilitates the study of the dynamics and spatial characteristics of hematopoiesis (Figure 5A) (26). Transgenic lines from *JAK1*^WT^ founders with high levels of expression in F1 generation were easily derived, but *JAK1*^GOF^ embryos had a poor survival rate and experienced multiple developmental abnormalities (Figure 5, B and C). To overcome this problem, we added ruxolitinib to the water for 24 hours starting at 6 hours postfertilization (hpf), which facilitated recovery of sufficient fish with *JAK1*^GOF^ expression to establish the line (*P* < 0.0001 for survival difference between ruxolitinib treated and untreated groups; Figure 5B). The toxicity of the *JAK1*^GOF^ transgene was not limited to the F0 embryos, since *JAK1*^GOF^ F1 embryos with high expression of the transgene also showed abnormal development (Figure 5, D and E).

### Increased myeloid-derived hematopoietic cells in zebrafish transgenic for human JAK1^GOF^ variant.

**Figure 6:**
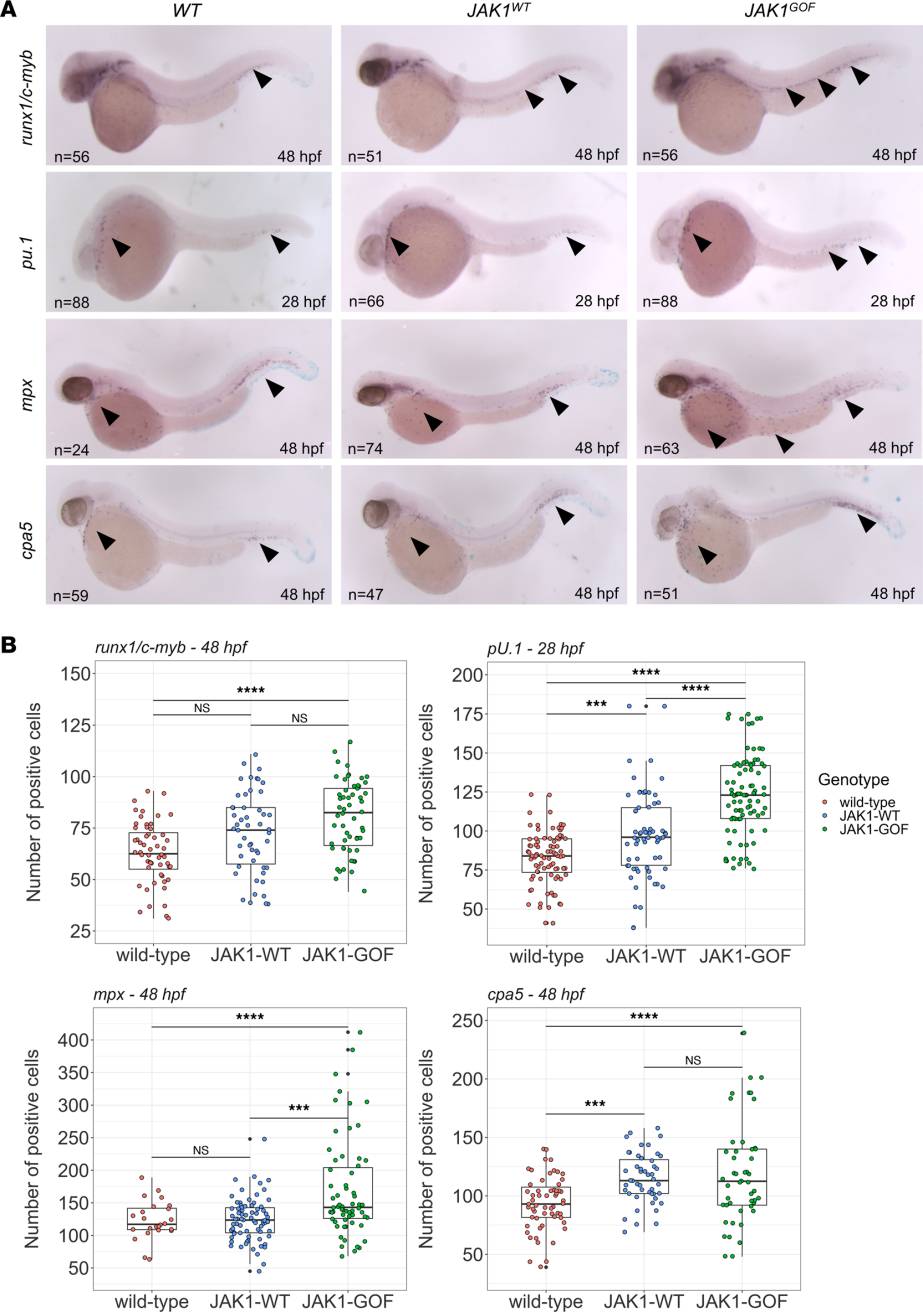
Characterization of the hematological phenotype of the human *JAK1*^WT^ and *JAK1*^GOF^ transgenic fish lines. (**A**) Whole-mount in situ hybridization (WISH) using digoxigenin-labeled RNA antisense probes for stem cells and differentiated myeloid cells in zebrafish embryos of *JAK1*^WT^ and *JAK1*^GOF^ transgenic genotypes. A panel of representative micrographs shows *runx1/c-myb* staining for hematopoietic stem cells at 48hpf, *pu.1* staining for early myeloid cells at 28 hpf, *mpx* staining for neutrophils at 48 hpf, and *cpa5* staining for mast cells at 48 hpf. Total numbers of imaged and quantified embryos are shown on the representative images, and arrowheads indicate main sites of marker expression. (**B**) Plots of marker-positive cell counts for *runx1/c-myb* at 48 hpf, *pu.1* at 28 hpf, and *mpx* and *cpa5* at 48 hpf. Each individual embryo count is indicated by a filled circle, and the box plot shows quartile distribution with whiskers covering 95% CI. Black circles denote location of outlier counts. One-way ANOVA was used to quantify the statistical differences between the groups. ***P ≤ 0.001; ****P ≤ 0.0001.

As vertebrates, zebrafish hematopoiesis is completed in 2 waves: a primitive wave (0 to approximately 28 hpf) where precursor erythroid and myeloid cells are generated, and a definitive wave (from approximately 36 hpf) where hematopoietic stem and progenitor cells (HSPCs) are generated and will create all the blood cell types in the adult fish (27). To characterize the effects of the *JAK1*^WT^ or *JAK1*^GOF^ transgenes on zebrafish hematopoiesis, we performed whole mount in situ hybridization (WISH) on embryos at 24, 28, 36, and 48 hpf. A battery of lineage-specific probes was used: *c-myb*/*runx1* (hematopoietic progenitor and stem cells), *pu.1* (myeloid progenitors), *mpx* (neutrophils), *cpa5* (mast cells), and *gata1* (early erythroid cells) (Figure 6 and Supplemental Figures 4–8).

The *JAK1*^GOF^ variant had a marked impact on zebrafish hematopoiesis. HSPCs were increased in *JAK1*^GOF^ transgenic fish at 36 and 48 hpf compared with nontransgenic *casper* (WT) (Figure 6 and Supplemental Figure 4). Myeloid progenitors (*pu.1*) were increased in *JAK1*^GOF^ compared with WT at 24 and 28 hpf (Figure 6 and Supplemental Figure 5). While *JAK1*^WT^ showed higher numbers of myeloid progenitors compared with WT at 28 hpf, this difference was further increased in the presence of *JAK1*^GOF^. Looking further downstream in the myeloid lineage, we observed increased neutrophils (*mpx*) at 36 and 48 hpf in *JAK1*^GOF^ compared with both the *JAK1*^WT^ transgenic strain and WT zebrafish (Figure 6 and Supplemental Figure 6); a similar increase of mast cells (*cpa5*) was observed in both transgenic fish at 48 hpf compared with WT zebrafish (Figure 6 and Supplemental Figure 7). Together, these data indicate an increase in myeloid-derived hematopoietic cells in zebrafish due to transgenic expression of human *JAK1*^WT^, which was further enhanced in the presence of the *JAK1*^GOF^. We also observed an increase of early erythroid cells at 24 hpf in the *JAK1*^GOF^ fish compared with WT and a trend with the *JAK1*^WT^ (*P* = 0.0378 and *P* = 0.0746, respectively; Supplemental Figure 8, A and B).

### Transcriptomic profiling of JAK1^GOF^ transgenic zebrafish.

**Figure 7:**
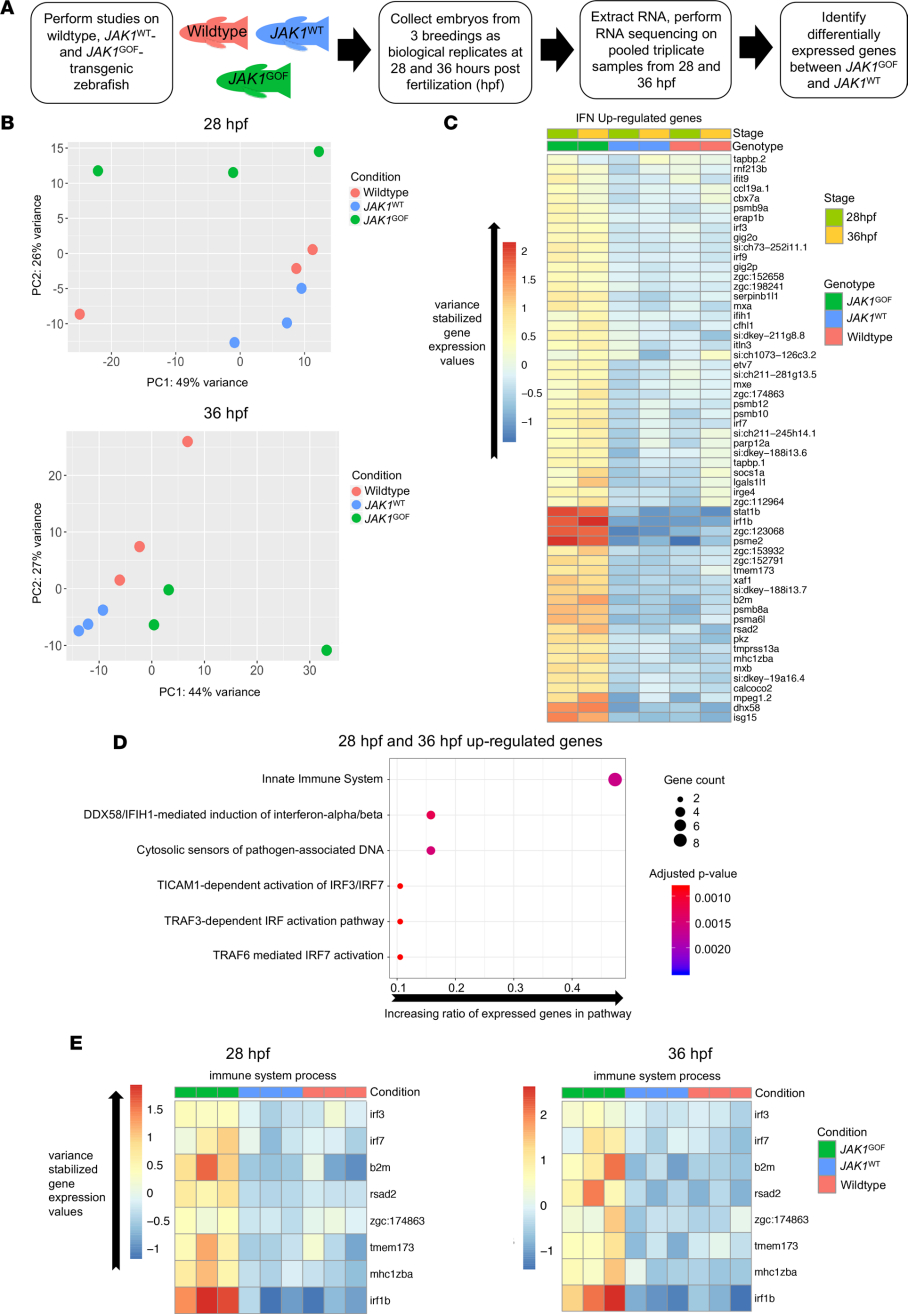
RNA-Seq of uninjected zebrafish and human *JAK1*^WT^ and *JAK1*^GOF^ transgenic zebrafish reveals a distinct transcriptomic pattern associated with *JAK1*^GOF^ (p.A634D). (**A**) Description of steps involved in the RNA-Seq experiment aimed at identifying genes specifically induced by JAK1-A634D (*JAK1*^GOF^) in zebrafish embryos. After performing standard sample collection and RNA-Seq steps, the main comparison at the analysis stage was between *JAK1*^WT^ and *JAK1*^GOF^ samples. (**B**) PCA of 28 and 36 hpf data sets consisting of WT, *JAK1*^WT^, and *JAK1*^GOF^ samples. The plots shown contain the first 2 components (PC1, PC2) and identify clear differences between *JAK1*^GOF^ samples and *JAK1*^WT^ or uninjected samples at 28 hpf. (**C**) Heatmap of average variance-stabilized gene expression values for IFN-stimulated genes that are present among the genes upregulated by *JAK1*^GOF^ in at least 1 stage analyzed (28 and 36 hpf) (58 genes) in all analyzed groups of samples. (**D**) Reactome Pathway analysis of genes upregulated by *JAK1*^GOF^ shows strong enrichment of immunity-related and DNA damage pathways. Gene ratio is the fraction of the genes in a pathway that were present in the input gene list. Gene count and *P* values associated with a pathway are indicated by the point size and its color. (**E**) Heatmaps of average variance-stabilized gene expression values for genes associated with the “immune system process” GO term in all analyzed groups of samples.

To generate further insight into the developmental impact of the *JAK1*^GOF^ variant, we analyzed the global transcriptome by performing RNA-Seq experiments at 28 and 36 hpf with 3 genotypes: WT, *JAK1*^WT^, and *JAK1*^GOF^ (3 groups each with 30 embryos per group) (Figure 7A). PCA and multidimensional scaling (MDS) were performed using gene-level read counts, and the first 2 components were plotted (Figure 7B and Supplemental Figure 9A). For the 28 hpf samples, PC2 or X2 components could cleanly separate the *JAK1*^GOF^ samples from WT zebrafish and *JAK1*^WT^ samples (Figure 7B).

### JAK1^GOF^ upregulates innate immune and IFN-stimulated genes in zebrafish.

To identify effects specific to *JAK1*^GOF^, further analysis directly compared *JAK1*^GOF^-differentially expressed genes to *JAK1*^WT^ (Figure 7, C–E). A greater-than 2-fold increase in expression of 152 and 211 genes among the *JAK1*^GOF^ group was observed at 28 and 36 hpf stages, respectively, of which 105 were common to both stages (Supplemental Figure 9B). Fewer genes were downregulated in the presence of the *JAK1*^GOF^ variant with 121 and 135 genes being downregulated at 28 and 36 hpf, respectively, among which 75 genes were shared (Supplemental Figure 9B). Reactome Pathway analysis of the 105 genes upregulated by *JAK1*^GOF^ at both 28 and 36 hpf demonstrated enrichment in innate immune and type 1 IFN signaling pathways (Figure 7D). Groups of genes were present in many pathways such as proteasome genes (*psmb8a*, *psme2*, *psmb9a*) and IFN pathway–related genes (*irf3*, *irf7*, *stat1b*, *socs3a*, *socs3b*). Similarly, GO analysis of upregulated genes at both 28 and 36 hpf identified terms related to immune system processes and responses to viruses and biotic stimuli (Figure 7E and Supplemental Table 5).

Given the role of JAK1 in IFN signaling (28), we explored in greater depth what fraction of upregulated genes can be accounted for by IFN-stimulated genes (ISGs) using a comprehensive list of ≥ 600 ISGs in zebrafish (29). We found an intersection of ISGs and all upregulated genes in *JAK1*^GOF^ zebrafish, which consists of 58 genes, thus accounting for about 22% of all upregulated genes. A heatmap of these genes in all samples clearly shows that these genes are only consistently upregulated in *JAK1*^GOF^ (Figure 7C). Taken together, *JAK1*^GOF^ likely exerts some of its impact via ISGs.

### Defining the core set of differentially expressed genes shared by the JAK1^GOF^ zebrafish and human models.

**Figure 8:**
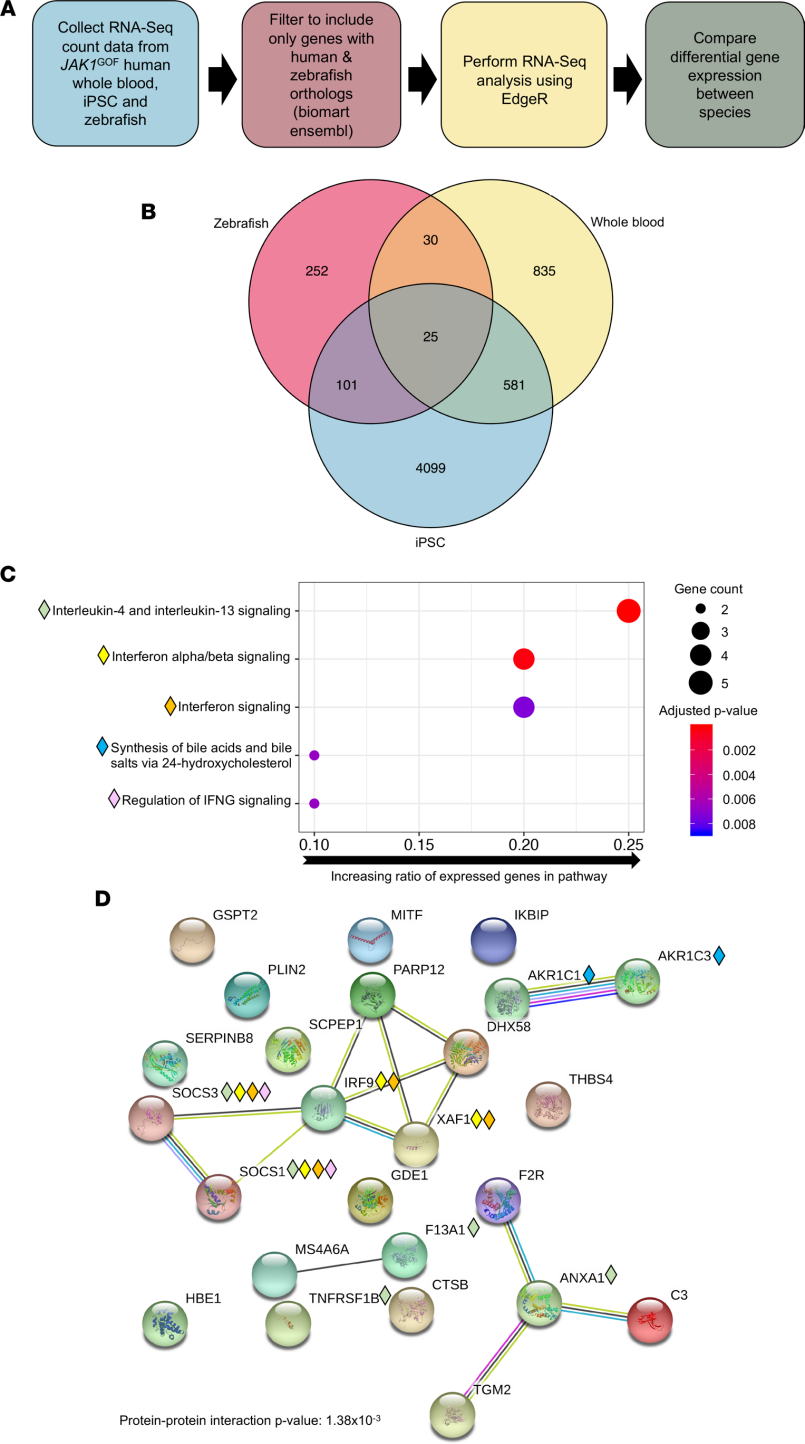
*JAK1*^GOF^ drives IL-4, IL-13, and IFN signaling across species. (**A**) Diagrammatic explanation of analysis pipeline for comparing differential gene expression between zebrafish, induced pluripotent stem cells (iPSC), and human whole blood. (**B**) Analysis of differentially expressed orthologous genes identified 25 genes that were common between human *JAK1*^GOF^ iPSCs, human *JAK1*^GOF^ whole blood, and *JAK1*^GOF^ transgenic zebrafish. (**C** and **D**) Reactome Pathway analysis and STRING protein-to-protein interactions of the 25 differentially expressed genes in *JAK1*^GOF^-affected patients, iPSCs, and transgenic zebrafish reveals significantly enriched protein-to-protein interactions, as well as IFN and IL-4/IL-13 signaling. The 5 most significantly upregulated Reactome Pathways are displayed in **C**. The *y* axis indicates Reactome Pathway, and the *x* axis corresponds to ratio of upregulated genes to the number of genes in each pathway; the size of each dot corresponds to number of genes identified. Color corresponds to the adjusted *P* value with red being more significant. The upregulated Reactome Pathway corresponding proteins are annotated in **D**, revealing the shared expression of SOCS proteins in both IFN and IL-4/IL-13 signaling.

To define the core set of differentially expressed genes, the human iPSC/whole blood and zebrafish gene expression data were then analyzed focusing solely on genes that have known orthologues between the 2 species. All differentially expressed genes with a FDR of less than 5% were compared between groups (Figure 8A). Twenty-five genes were differentially expressed in human *JAK1*^GOF^ whole blood, iPSC, and *JAK1*^GOF^ transgenic zebrafish (Figure 8B). Reactome Pathway analysis revealed IL-4/IL-13 signaling as the most significantly enriched core pathway among shared differentially expressed genes, followed by IFN-α/β signaling (30, 31) (Figure 8C). Functional enrichment analysis of the differentially expressed genes shared among all 3 models revealed significantly more interactions than expected (Figure 8D), with a protein-to-protein interaction enrichment *P* value of 1.38 × 10^–3^ (30). While genes unique to both the IL-4/IL-13 and IFN-α/β pathways were identified, *SOCS1* and *SOCS3* are present in both these pathways (Figure 8, C and D). Given the established role of JAK1 in IL-4/IL-13 and IFN signaling, this finding supports the underlying validity of these model systems.

## Discussion

Studying IEIs that cause allergic inflammation is a powerful strategy to improve our understanding of the pathophysiology underlying common allergic conditions. Moreover, uncovering the molecular basis of IEIs creates the potential for “precision medicine,” where treatments can be selected that target the specific pathway impaired by the genetic defect (32–34). At the level of the broader population, defining the mechanisms underlying severe monogenic allergic disease is anticipated to uncover new targets for preventing and treating common allergic disorders (35).

In this study, by leveraging a combination of human and zebrafish experimental systems to model the human germline *JAK1*^GOF^ (p.A634D) variant, we highlight several key mechanisms by which overactive JAK1 signaling leads to abnormal hematopoiesis and allergic immune dysregulation, particularly Th skewing and enhanced myelopoiesis. We provide evidence that this is being driven by type 2 inflammation, demonstrated by IL-4/IL-13 and Th2 gene expression signatures and clinical allergic disease that dramatically responds to Jakinib therapy. This is particularly surprising, given the role of JAK1 in IFN signaling, which is traditionally associated with manifestations of type 1 IFNopathies including severe neurologic and vasculitic/chilblain-like skin lesions (36). Perhaps most importantly, these experimental models were consistent with many of the clinical features seen in patients carrying the *JAK1*^GOF^ variant and provide mechanistic evidence for their long-term response to oral Jakinib therapy.

Using independent analytical tools, we demonstrate that patients with enhanced JAK1 signaling have an increase in various Th lineages — most notably, Th2 cells. This finding suggests that *JAK1*^GOF^ drives T cell activation and differentiation, and skewing toward Th2 may contribute to the observed eosinophilia. Interestingly, following ruxolitinib treatment, the Th1 gene expression signature increased. The significance of this is unknown, however, given the counter-regulatory effects of Th2 and Th1 genes under certain conditions, it is possible that upon treatment with ruxolitinib, the inhibition of Th2 signaling led to a transient increase in expression of Th1-associated genes. This finding warrants additional study. While our data generated by flow cytometry, RNA-Seq, and primary cell culture experiments all demonstrated Th2 skewing in *JAK1*^GOF^ primary cells, we did face challenges inherent in studying a single family. Specifically, since 2 of the 3 patients were already on treatment with ruxolitinib, only 1 patient (II-2) was available for the T cell phenotypic analysis. Subsequently, II-2 initiated treatment with a selective JAK1 inhibitor upadacitinib, and therefore, the studies assessing T cell memory and cytokine production could not be repeated.

Ten months after patient II-2 started taking upadacitinib, intracellular cytokine staining showed a modest decrease in proportions of IL-4–, IL-17–, and IFN-γ–secreting cells compared with pretreatment. This is in contrast to the observation in ruxolitinib-treated patients III-1 and III-2, where a significant decrease in the Th2 gene expression signature was observed following treatment with ruxolitinib. Our findings align with patient II-2’s clinical response to upadacitinib; although her atopic dermatitis and itch improved, she did not share the dramatic clinical improvement that III-1 and III-2 experienced from ruxolinitib. Possible explanations for the less-dramatic response to selective JAK1 inhibition with upadacitinib in II-2 compared with JAK1/2 inhibition with ruxolitinib in III-1 and III-2 include the following: the upadacitinib dose was insufficient to fully counteract the enhanced JAK1 signaling caused by the *JAK1*^GOF^ variant, or selective JAK1 inhibition did not address transactivation of other JAK family kinases (such as JAK2 or TYK2) by the JAK1 pseudokinase (4). Alternatively, the modest changes to intracellular cytokine staining pre- and postupadacitinib, despite improvement in atopic dermatitis, could imply that JAK1-mediated allergic immune dysregulation is driven by non–T cells. Indeed, the impact of other cells, such as innate-like lymphocytes (ILCs), on JAK1-mediated atopy needs to be investigated. Since upadacitinib recently achieved regulatory approval for treatment of atopic dermatitis, it is important to further study the impact of dosing on T cell immunophenotype and clinical response.

Our T cell findings align with previous *JAK1*^GOF^ animal models. The *Spade* R878H *JAK1*^GOF^ mice primarily manifested with dermatitis and Th2 inflammation, with increased IFN-γ also noted; similarly, our patients had the most pronounced expansion of Th2 cells and IL-4, with trends toward enhanced Th1 and Th17 compartments, and a pronounced increase in IFN-γ production. This finding of multiple cytokine axis involvement has been described in allergic diseases such as atopic dermatitis; patients can display Th2-predominate inflammation complicated by additional Th1 and Th17 cytokine axes, influenced by timing in disease course, age, and other factors (37). As JAK1 functions downstream of cytokines important in differentiation of many Th subsets, it is not surprising that enhanced JAK1 signaling may translate to increased effector responses through multiple cytokine axes. Our patient’s increased IFN-γ production in CD4^+^ T cells also aligns with the biallelic JAK1 LOF phenotype that inversely shows deficient IFN-γ and susceptibility to mycobacterial infection (38).

Using both human and zebrafish model systems, we demonstrate that *JAK1*^GOF^ drives myelopoiesis. This was first observed in zebrafish models as an increase of HSPC, followed by an increase of the common myeloid progenitors and by the granulocyte-monocyte progenitor. Zebrafish do possess eosinophils, but to date, their identification and isolation has only been in adult fish, since eosinophils are a rare population in the absence of a helminthic stimulus (10). The absence of a specific marker for eosinophils in larvae prevented us from evaluating their numbers using WISH in our zebrafish model. However, we did observe an increase in neutrophils and mast cells, which are cells that are closely related to eosinophils, with roles in innate and adaptive immunity and allergic inflammation, respectively (39). Although we did not directly identify why eosinophils are disproportionately affected in *JAK1*^GOF^, we show that this condition drives myelopoiesis and type 2 inflammation, and eosinophilia has been observed in other patients carrying *JAK1*^GOF^ variants (4). In the human *JAK1*^GOF^ iPSCs, we saw increased expression of the IL-33 receptor gene (*IL1RL1*), which is a marker of early eosinophil precursor commitment (12). IL-33 is an important driver of eosinophilia, promoting both eosinophil development and survival, and a chromosomal duplication encompassing the IL-33 gene was recently identified in a patient with hypereosinophilia and atopy (40). In Th2 cells, IL-33R expression is dependent on both GATA3 and STAT5 (41). Future work will aim to confirm the role of *JAK1*^GOF^ in promoting IL-33R expression on hematopoietic cell precursors and whether this drives eosinophil lineage commitment and resulting eosinophilia.

While it is possible that genetic factors independent of JAK1 may contribute to the allergic skewing observed in patient-derived *JAK1*^GOF^ iPSCs, the shared gene expression signatures concurrently observed in the *JAK1*^GOF^ transgenic zebrafish support that JAK1 is driving the immunologic abnormalities observed in our iPSC model. While we are confident in our findings, further confirmation will arise through the identification and study of more patients with *JAK1* variants such as S703I GOF and P733L/P832S loss of function (4). Our findings suggest that elevated levels of active JAK1 promote a skewing toward the myeloid lineage during hematopoiesis. This may ultimately give rise to enhanced eosinophil production, contributing to allergic inflammation.

By evaluating the orthologous genes differentially expressed in both *JAK1*^GOF^ humans and *JAK1*^GOF^ transgenic zebrafish, we were able to identify the most important genes and pathways impacted by enhanced JAK1 signaling. RNA-Seq analysis identified a number of differentially expressed genes in both zebrafish and humans that are involved in the antiviral response and in IL-4/IL-13 signaling. These findings highlight JAK1’s central role in both IFN and type 2 inflammatory signaling, and they support how dysregulated JAK1 signaling can lead to severe allergic inflammation that improves with Jakinib treatment. As was observed in the *Spade* *JAK1*^GOF^ mouse model (5), both zebrafish and humans carrying *JAK1*^GOF^ variants demonstrated increased expression of *SOCS1* and *SOCS3*. This is notable because overexpression of zebrafish *socs1* generates a phenotype similar to *JAK1*^GOF^ zebrafish with enhanced myelopoeisis (42), suggesting that overexpression of regulatory molecules in the setting of dysregulated cytokine signaling can also disrupt cell homeostasis and contribute to abnormal skewing of hematopoietic cell populations.

The differentially expressed signaling molecules and cytokines/cytokine receptors identified in human *JAK1*^GOF^ whole blood and iPSCs suggest how dysregulated JAK1 signaling could lead to abnormal skewing toward an exaggerated type 2 response early in life. The increased expression of TL1A (*TNFSF15*), *CSF1*, *CCL25*, *TSLP*, and IL-33R (*IL1RL1*) in *JAK1*^GOF^ iPSCs suggests that *JAK1*^GOF^ orchestrates early life allergic inflammation through innate cells and ILCs, such as ILC2s, whose key receptors were expressed in *JAK1*^GOF^ whole blood (IL-25R, IL-33R, and IL-2RA). Given the particularly severe manifestations of *JAK1*^GOF^ during fetal development (as demonstrated by poor intrauterine growth, hepatic cysts, respiratory distress at birth) and in early infancy where patients III-1 and III-2 experienced the most severe increases in eosinophil counts, it is possible that ILCs could be important drivers of the atopic immune dysregulation in early life (43, 44). This has important implications when considering the “early window” of allergic disease prevention, and it will be a future focus of research (45). TL1A is a ligand for lymphocytes that has been implicated in inflammation and fibrosis affecting the liver, skin, and mucosal sites, as well as eosinophilic asthma via ILC2-mediated IL-5 production (46, 47). The increased expression of TL1A in patients with *JAK1*^GOF^ is striking and suggests this as a possible mechanism for their multisystem inflammatory disease and liver injury. Furthermore, enhanced JAK1 signaling impacts numerous cell types, including those of nonhematopoietic lineage that can also play important roles in the pathophysiology of allergic disease, such as the epithelial barrier. The nonimmune manifestations observed in our patients also suggest the importance of JAK1 in a variety of tissues. One limitation of this study is that the focus was on cells of hematopoietic origin and did not include biopsies of skin or other nonhematopoietic tissue aside from the iPSC and zebrafish work. Upregulation of genes encoding for TSLP and TL1A, which are expressed in nonimmune cells, supports further investigation into the nonhematopoietic drivers of JAK1-mediated allergic inflammation.

In addition to clarifying the mechanisms underlying JAK1-mediated allergic inflammation using these models, we looked at their clinical implications and the impact of ruxolitinib in the affected family. *JAK1*^GOF^ patients experienced rapid and sustained improvements in growth and allergic immune dysregulation on ruxolitinib. Although improved, patient III-1 continues to have significant airway hyperresponsiveness and moderate eosinophilia. Patient III-2, who was started on ruxolitinib at less than 2 years of age, has avoided developing food or environmental allergies, has had only mild respiratory symptoms, and has minimal evidence of hepatic inflammation. This demonstrates that early disease modification through medications that normalize skewed signaling pathways may influence the natural history of allergic disease in predisposed individuals. There were no major adverse events associated with ruxolitinib, aside from anemia, which improved with dosage adjustments. This experience somewhat alleviates concerns related to infectious risks associated with ruxolitinib use and potential impact on growth in a pediatric population. These conclusions are limited by the small sample size, as this is the only published familial case of *JAK1*^GOF^. It is also possible that patients whose baseline JAK1 signaling is lower than that mediated by a strong *JAK1*^GOF^ pathogenic variant as observed in this family may experience a different side effect profile.

In conclusion, we show that heterozygous *JAK1*^GOF^ leads to an allergic immune dysregulatory syndrome causing enhanced myelopoiesis and type 2 inflammation. Zebrafish, human iPSC, and human blood samples effectively demonstrated the effects of enhanced JAK1 signaling on hematopoiesis and immune function; these tools can be used in future studies to probe further into the impact of JAK1 on health and disease. This work highlights the promise of JAK1 as a therapeutic target in allergic inflammation and the potential for disease-modifying effects during the “early window” of allergic prevention.

## Methods

### Pharmacotherapy.

Patient III-2 was trialed on prednisolone at a dose of 2 mg/kg/d. After no response to therapy and discovery of the *JAK1*^GOF^ variant, patients III-1 and III-2 were initiated on ruxolitinib (Jakafi; Incyte) treatment at a dose of 50 mg/m^2^/dose administered twice daily. Dosing was informed based on a phase I study of ruxolitinib for children with malignancy and myeloproliferative disorders (48). Dosing was titrated to effect based on symptoms such as pruritus and feeding intolerance, with dose increases limited by the side effect of anemia.

### Generating iPSC lines.

PBMCs were isolated from *JAK1*^GOF^ patient whole blood using Ficoll-Hypaque density-gradient centrifugation (400*g* for 30 minutes at room temperature). PBMCs underwent erythroid progenitor expansion by culturing in StemSpan II and Erythroid Expansion Supplement (STEMCELL Technologies) for 7 days. Expanded progenitors were then reprogrammed into pluripotency using Sendai virus, a nonintegrating RNA viral vector carrying the transcription factors *OCT4*, *SOX2*, *KLF4*, and *cMYC*. Treated cells were plated onto matrigel on day 3 after reprogramming and transitioned to ReproTeSR medium (STEMCELL Technologies) on days 5–7. iPSC colonies were picked on days 12–21 and underwent successive passaging. Sendai virus was removed by heat shock on passage 10. The XY control iPSC line was also generated by the authors (49). Both the *JAK1* ^GOF^ and XY control iPSC lines demonstrated normal karyotype and pluripotency confirmed by pluripotency marker analysis (Supplemental Figure 10).

### Culturing of iPSC lines.

iPSC lines were maintained in StemFlex medium and passaged every 3–7 days using ReLeSR (STEMCELL Technologies) into StemFlex medium containing 10 μM ROCK inhibitor. Media was replaced the day following passaging with StemFlex without ROCK inhibitor. iPSC pluripotency and normal karyotype were confirmed prior to performing experiments, and PCR sequencing confirmed that the *JAK1*^GOF^ iPSC carried the *JAK1* c.1901C>A variant (Supplemental Figure 10). DNA extracted from *JAK1* ^GOF^ iPSCs was amplified by PCR using the following primers (5′–3′): GGA TCT GGT GCA GGG CGA GC (forward) and GTC GCG GAC ACA GAC GCC AT (reverse).

### EB formation and myeloid differentiation.

*JAK1*^GOF^ and XY control iPSC cells were split using ReLeSR (STEMCELL Technologies) onto ultra-low adhesion plates. EBs were grown in culture with bFGF-free stem cell medium containing 300 ng/mL FLT3 (PeproTech), 300 ng/mL SCF (PeproTech), 50 ng/mL BMP-4 (R&D Systems), 50 ng/mL G-CSF (R&D Systems), 10 ng/mL IL-3 (PeproTech), and 10 ng/mL IL-6 (R&D Systems) as described previously (50, 51). RNA was extracted from collected EBs on day 7 using QIAGEN RNeasy Plus Mini Kit. After 16 days, EBs were dissociated into single cells using 1 mg/mL Collagenase B, nonenzymatic cell dissociation buffer (Thermo Fisher Scientific) and mechanical sheering. Cells were then plated onto methylcellulose (MethoCult H4434 containing SCF, EPO, IL-3, and GM-CSF; STEMCELL Technologies) and cultured for 14 days (50). Throughout the experimental period, samples were cultured in 21% O_2_, 5% CO_2_ at 37°C.

### Th subset phenotyping by chemokine receptor analysis.

PBMCs were isolated and incubated overnight in RPMI complete medium at a concentration of 1 × 10^6^ cells/mL. Cells were then washed with PBS twice and subsequently stained with a fixable viability stain and monoclonal antibodies to the following human proteins: CD3 (catalog 641406), CD4 (catalog 552838), CD8 (catalog 562429), CCR4/CD194 (catalog 557864), CXCR3/CD183 (catalog 557185), CCR10 (catalog 564771), and CCR6/CD196 (catalog 564479) (all from BD Biosciences). Cells were washed twice in FACS buffer and subsequently analyzed by flow cytometry on a BD LSR II machine. Staining for memory T cells involved staining with a fixable viability stain and monoclonal antibodies to the following human proteins: CD3 (catalog 641406), CD4 (catalog 552838), CD45RA (catalog 560675), and CCR7 (catalog 150503) (all from BD Biosciences), washed twice with FACS buffer, acquired by flow cytometry on a BD LSR II machine and analyzed using FlowJo software.

### Activation, expansion, and intracellular cytokine staining of primary human T cells.

PBMCs were isolated from whole blood by means of centrifugation over a Ficoll gradient at 400*g* for 30 minutes at room temperature and stimulated in complete medium containing IMDM (HyClone), supplemented with 10% FBS, 2 mM L-glutamine, 1× nonessential amino acids (all from Thermo Fisher Scientific), and 57 μM β-mercaptoethanol in the presence of anti-CD2/CD3/CD28 beads (Miltenyi Biotec). Cells were split every 2–4 days with medium containing human recombinant IL-2 (40 U/mL) to maintain a cell concentration of 5 × 10^5^/mL. After 7 days of expansion to generate sufficient cells for experimentation, T cells were incubated with phorbol 12-myristate 13-acetate (PMA), ionomycin, and GolgiStop (BD Biosciences), according to the manufacturer’s instructions in complete medium for 4 hours. Cells were then fixed and permeabilized using BD Cytofix Fixation Buffer BD Perm/Wash Buffer, respectively (both from BD Biosciences), and stained with a fixable viability stain and monoclonal antibodies against the following human proteins: CD3 (clone 641406), CD4 (clone SK3), IFN-γ (clone Β27), IL-17 (clone N49-653), IL-4 (clone MP4-25D2), and IL-9 (clone MH9A3) (all from BD Biosciences). Data were collected with an LSR II cytometer (BD Biosciences) and analyzed using FlowJo software.

### Cytokine gene expression analysis following in vitro stimulation and JAK inhibitor treatment.

The expanded T cells were incubated with complete T cell medium containing recombinant human IL-2 (100 U/mL) (PeproTech), recombinant human IL-2 (100 U/mL) with ruxolitinib (1 μM) (Selleckchem), or dimethyl sulfoxide (DMSO) vehicle for 4 hours. RNA was extracted using the RNeasy Mini Kit (QIAGEN). RNA was reverse transcribed to cDNA using the iScript reverse transcription supermix for qPCR (Bio-Rad). qPCR of *IL4* and housekeeping gene *RPL13A* were performed using 2× SYBR Green master mix and a ViiA7 Real-Time PCR System (Applied Biosystems). Gene expression was measured using the 2^–ΔΔCt^ (Livak) method.

### Whole blood and iPSC RNA-Seq.

Whole blood was collected by venipuncture from HCs and from *JAK1*^GOF^ patients before and between 2 and 7 months after treatment with ruxolitinib. RNA was extracted and quality assessed using the Agilent 2100 Bioanalyzer. Messenger RNA was converted to complementary DNA, amplified, and sequenced to a depth of at least 15 million reads using the Ion Torrent library kits and the Ion Proton next generation sequencing system (Thermo Fisher Scientific) at the UBC sequencing core at the Djavad Mowafaghian Centre for Brain Health. Raw sequence reads were aligned to the human genome (hg19) with a 2-step protocol using Tophat2 followed by Bowtie2 (52, 53). Read counts were quantified using HTSeq-count.

RNA was extracted from *JAK1*^GOF^ and XY control iPSC EBs on day 7 of myeloid differentiation. Sample quality control was performed using the Agilent 2100 Bioanalyzer. Qualifying samples were then prepped following the standard protocol for the NEBNext Ultra II Stranded mRNA (New England Biolabs). Sequencing was performed on the Illumina NextSeq 500 with Paired End 42 × 42 bp reads. Demultiplexed read sequences were then aligned to the reference sequence using STAR single aligner software package (54). Assembly and differential expression was estimated using Cufflinks through bioinformatics apps available on Illumina Sequence Hub (55).

### GO and reactome pathway analysis.

Enrichment for GO terms was performed using GOrilla using 2 unranked lists for target and background genes (56). All types of GO terms were reported in Supplemental Tables 2 and 5, and selected groups of genes corresponding to 1 or several related GO terms were chosen for plotting heatmaps. Reactome Pathway analysis was performed after determining differentially expressed genes by edgeR using the ReactomePA Bioconductor package (57).

### RNA-Seq bioinformatics analysis.

Differentially expressed genes were identified using edgeR Bioconductor library (58), using the 2-fold up- or downregulation thresholds and FDR ≤ 0.05 as parameters for both the human and zebrafish RNA-Seq analysis. PCA and MDS plots were produced using DESeq2 library (59). Heatmaps were produced using pheatmap package (60) after performing variance stabilizing transformation using a function from DESeq2 library. Th cell subsets were analyzed from whole-blood RNA-Seq data by performing PCA of Th1, Th2, and Th17 gene expression signatures obtained from the literature (20). The first principal component values were compared between patients before and after treatment and to unrelated HCs.

### Zebrafish husbandry, embryo collection, and embryo staging.

All strains of zebrafish used for this project were generated by the authors and were maintained according to standard protocol (61). Embryos are collected and grown in E3 embryo media at 28°C. When embryos lacking pigmentation were required, we treated them with an embryo medium containing 0.003% N-phenylthiourea (PTU) (Sigma-Aldrich, P7629). Embryos were dechorionated by incubating them for 10 minutes in 1 mL of egg water after adding 50 μL of 10 mg/mL stock solution of Pronase (Roche Applied Science, 10165921001). Embryos were developmentally staged according to standard protocol. *Casper* zebrafish (*mitfa*^w2/w2^; *mpv17*^a9/a9^) were used as WT controls.

### JAK1 transgenic line creation.

We amplified *JAK1*^WT^ and *JAK1*^A634D^ cDNA PCR products from pCMV6-Entry vectors using Q5 (NEB, M0492S) with PacI-JAK1_for (ggacttaattaagccaccatggctttctgtgctaaaatg) and AscI-JAK1_rev (gatcggcgcgccaaccttatcgtcgtcatcct) primers (5′–3′). We then inserted these PCR products into pCS2+MCS-P2A-sfGFP vector (Addgene, 74668) using digestion with PacI (NEB, R0547S) and AscI (NEB, R0558S) enzymes and standard cloning techniques. The resulting pCS2+JAK1-WT-P2A-sfGFP and pCS2+JAK1-A634D-P2A-sfGFP vectors were used to amplify the JAK1-WT/A634D-P2A-sfGFP inserts using Q5 with PacI-JAK1_for and sfGFP_rev (ttatttgtagagctcatccatg) primers (5′–3′). These PCR products were then inserted into pME_TA vector, as described by the developers of the vector system (62). The final pME- JAK1-WT-P2A-sfGFP and pME- JAK1-A634D-P2A-sfGFP vectors were LR recombined with *p5E-ubi* (63) *p3E-pA* and *pDestTol2_cry:ECFP* (Tol2 kit; ref. 64) using LR Clonase Plus II enzyme mix (Thermo Fisher Scientific, 12538120) according to manufacturer’s instructions; they were transformed into TOP10 cells and screened by digestion and sequencing. The final ubi-JAK1-WT/A634D–P2A-sfGFP-pA zebrafish transgenes were then injected into 1-cell stage zebrafish as part of an injection mix containing 20 ng/μL transgenic plasmid and 35 ng/μL pCS2FA transposase mRNA as described previously (64). These transgenic lines have allowed us to express ubiquitously both the WT (*JAK1*^WT^) and A634D (*JAK1*^GOF^) mutant variants of the human *JAK1* gene cDNA fused to the superfolder GFP (sfGFP) gene via a cotranslational cleavage peptide P2A. After observing decreased embryo viability in *JAK1*^GOF^ zebrafish with high expression of the transgene, embryos were treated with 1 μM ruxolitinib in fish water (Cayman Chemical, 941678-49-5) at 6 hpf for 24 hours to facilitate survival.

### WISH assays.

The probes used in the study were generated from the following plasmids with the restriction enzymes used for linearization and Addgene accession numbers include the following: *cmyb* (pBK-CMV-cmyb; BamHI; Addgene, 191804*)*, *cpa5* *(*pBK-CMV-cpa5; EcoRI; Addgene, 191805), *mpx* (pBK-CMV-mpx; EcoRI; Addgene, 191826), *pU.1* (pBK-pu1; SacI; Addgene, 191827), *gata1a* (pBSK-gata1a; SacI; Addgene, 191828), *lcp1* (pBSK-lcp1; EcoRI; Addgene, 191829), and *runx1* (pBSK-runx1; HindIII; Addgene, 191830). The probes were synthesized from 1 μg of the linear plasmid templates using T7 RNA polymerase (Thermo Fisher Scientific, EP0111), 10× DIG RNA labeling mix (MilliporeSigma, 11277073910), and Protector RNAse inhibitor (Roche, 3335399001) (1 μL/20 μL reaction) for 4 hours at 37°C and purified using the NucAway Spin Columns (Thermo Fisher Scientific, AM10070). WISH assays were performed on embryos fixed overnight in 4% paraformaldehyde (PFA) in PBS at 24, 28, 36, and 48 hpf, according to the previous protocol with the following modifications (65). Embryos at 24 and 28 hpf were permeabilized with 1 μg/mL proteinase K solution for 10 and 15 minutes, respectively. Embryos collected at 36, 48, and 144 hpf were incubated with 10 μg/mL proteinase K for 5, 10, and 30 minutes, respectively. The staining step was done with BCIP (Roche, 11383221001) and NBT (Roche, 11383213001) reagents diluted in the staining buffer. The stained embryos were then fixed in 4% PFA for 30 minutes, washed in PBST, and embedded into 80% glycerol for imaging. Images were taken on a CCD Zeiss Axiocam 506 color camera attached to a stereomicroscope with LED epi-illumination (Carl Zeiss, SteREO Discovery V20). Micrographs are representative of at least 2 independent trials with at least 15–20 embryos per genotype.

### WISH scoring.

For all probes, except *gata1*, the positive cells were manually counted using Fiji (66). For *gata1* that produces staining with no discernible single cells, counting was not possible. Therefore, we used a training set of 3 images, and then we proceeded to evaluate the rest of the samples using Ilastik and CellProfiler (67).

### Zebrafish RNA-Seq.

RNA was quantified and integrity was analyzed on Agilent Tape Station. Samples with RNA integrity number of ≥ 8 were selected for library preparation. Poly-A enrichment was performed from 20 μg of total RNA using Dyna beads mRNA direct micro Kit (61021, Thermo Fisher Scientific). In total, 100 ng of poly-A enriched RNA was fragmented using RNase III and purified using magnetic bead cleanup module (Ion Total RNA-Seq Kit v2, Thermo Fisher Scientific, 4475936). Yield and size distribution of the library was analyzed on Agilent Tape Station using D1000 screen tape. Barcoded library was equally pooled and amplified onto Ion Sphere Particles (ISPs) supplied by Ion Pi HiQ OT2 kit (Invitrogen). ISPs enriched with template library were loaded onto Ion PI chip V3 and sequenced on Ion Proton from Thermo Fisher Scientific.

The raw reads had the adapters “ATCACCGAC” removed and filtered by the quality of 20 with the Trim Galore (v0.4.4). The reads passed through the 2-step mapping for RNA-Seq data from Ion proton — first with STAR (v2.7) (54) and then mapping the unmapped reads from the first alignment using Bowtie 2 (68). The reference genome used in both mapping steps was the Genome Reference Consortium Zebrafish Build 11 (danRer11). The 2 mapped files were merged with Picard tools (v2.5.0). The counts were extracted using HTSeq (V0.9.1) (69). The zebrafish RNA-Seq data are available under GSE142599.

### Comparison of differential gene expression between JAK1^GOF^ zebrafish and humans.

In order to compare differential gene expression between zebrafish and human RNA-Seq data, a list of orthologous zebrafish gene names was generated by inputting the list of genes whose expression was quantified from human whole blood into the Ensembl biomart tool (70). RNA-Seq count data derived from both human whole blood and zebrafish models was filtered to only include genes with human and zebrafish orthologues. A FDR parameter of ≤ 0.05 was used to identify differentially expressed genes between species using the EdgeR Bioconductor library (58). Protein-to-protein interactions and relevant pathways were analyzed using STRING and Reactome, respectively (30, 31).

### Statistics.

All analyses were performed using Prism and Rstudio. Statistical analysis was performed using ANOVA (1- or 2-way as noted in text) or 2-tailed unpaired *t* test with Welch’s correction. Bonferroni multiple-comparison correction was used when applicable. RNA-Seq analysis performed as described above under “RNA Sequencing Bioinformatics Analysis.” A *P* value less than 0.05 was considered significant.

### Study approval.

This study received research ethical approval from the IRBs at the University of British Columbia and Dalhousie University. The Dalhousie University Animal Care Committee approved this study under protocol no. 17-038. Written informed consent was provided by participants and, in the case of minors, by their parents.

## Author contributions

CMB, ACS, SVP, APD, YL, FO, KLDB, RJR, and M Sharma performed the experiments and analyzed data. AS, SM, LD, and KES contributed to RNA-Seq data analysis. DD contributed to data statistical analysis. KGW, LODB, KD, and LDN provided training and guidance on iPSC modeling and myelopoiesis. SV and KES provided the BM aspirate slides and interpretation. JJP and KT provided guidance and reagents used for T cell activation and expansion. MLM, SV, M Seear, JR, RAS, and MCKH provided clinical patient care and contributed to manuscript review and preparation. FCL, JNB, and SET supervised experiments and contributed to manuscript review and preparation. CMB, ACS, and SVP contributed equally to the data production and are credited as co–first authors. CMB was chosen as the first name because she wrote the largest fraction of the manuscript; ACS was chosen as the second name because she contributed to writing and performed the zebrafish experiments; and SVP was chosen as the third name because he performed the zebrafish RNA-Seq analysis and assisted in graph generation and writing.

## Supplementary Material

Supplemental data

## Version 1 12/22/2022

Electronic publication
