# Supplementary material for: Human JAK1 gain of function causes dysregulated myelopoeisis and severe allergic inflammation
Source: JCI Insight. 2022 Dec 22;7(24):e150849. doi: 10.1172/jci.insight.150849 (PMC9869972; doi:10.1172/jci.insight.150849)
Supplement: Supplemental data [file jciinsight-7-150849-s030.pdf]

SUPPLEMENTAL DATA AND FIGURES

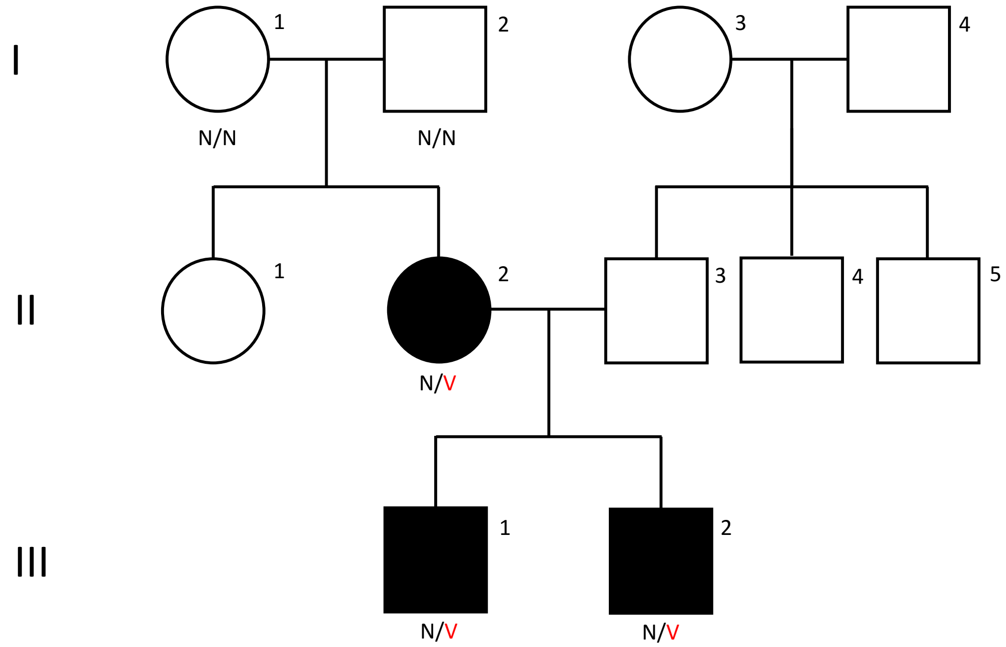

Supplemental figure 1: Pedigree of family carrying the *JAK1* c.1901 C>A nucleic acid substitution, corresponding to p.A634D. Affected individuals identified by black circle and squares are heterozygous for the *JAK1* c.1901 C>A nucleic acid substitution (V indicating variant and N indicating normal sequence). The affected mother's parents have normal biallelic *JAK1* sequencing.

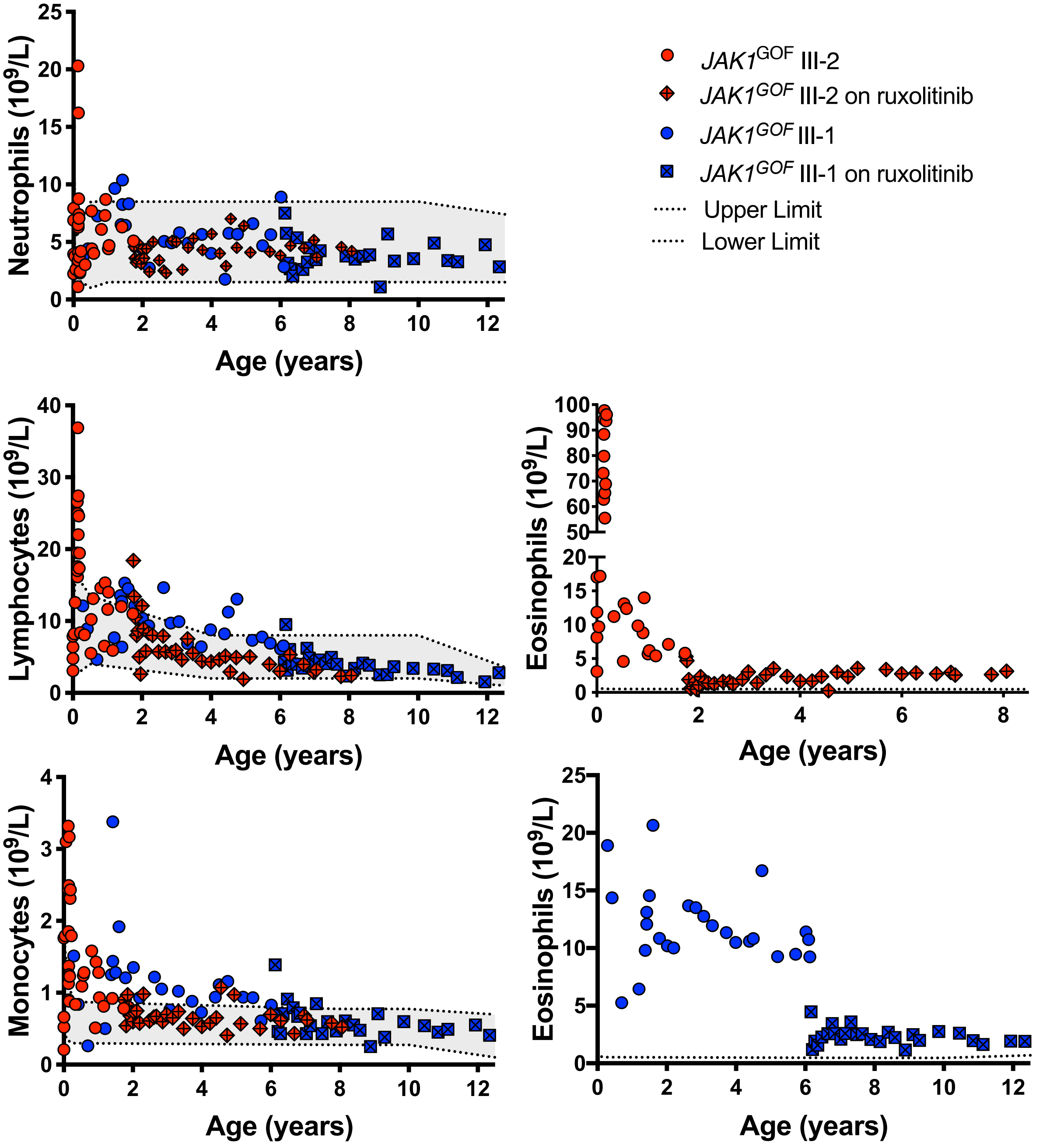

Supplemental Figure 2: Elevations in all white blood cell types in *JAK1*^{GOF}. White blood cell differential reveals increased numbers of neutrophils, lymphocytes, monocytes and eosinophils in *JAK1*^{GOF} patients. Ruxolitinib therapy normalized neutrophil, lymphocyte and monocyte numbers, while eosinophil counts dramatically improved however remain above the normal upper limit (0.5×10^9/L).

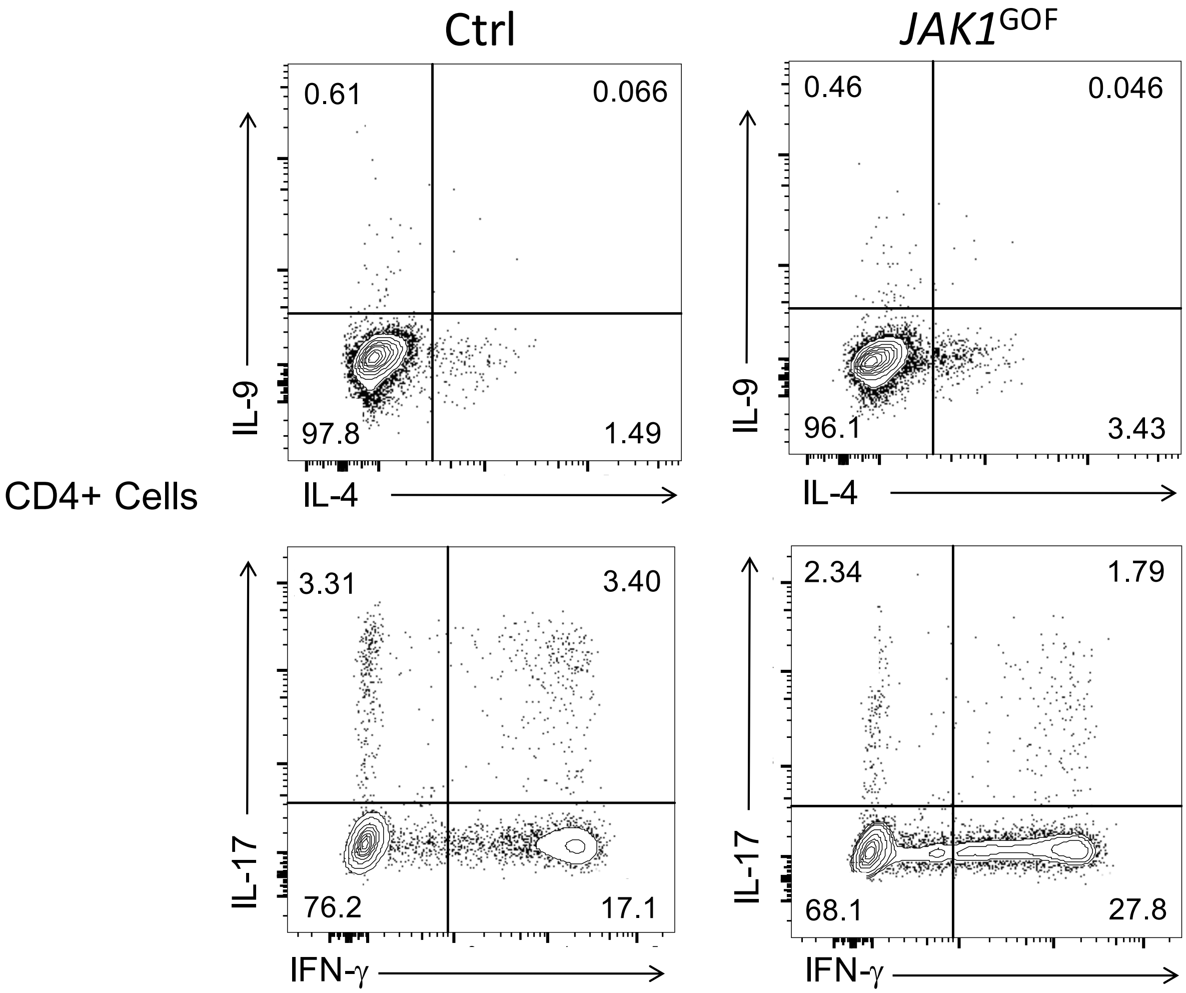

Supplemental Figure 3: Modest decrease in frequency of of IL-4, IFN-γ and IL-17 secreting *JAK1*^{GOF} CD4^{+} T cells after treatment with upadacitinib compared to pre-treatment values. The frequency of IL-4 and IFN-γ secreting cells in the *JAK1*^{GOF} patient remained elevated, however, in comparison to controls.

Supplemental table 1: Reactome pathway analysis of genes upregulated in both *JAK1*^{GOF} whole blood and iPSC

ID	Description	BgRatio	p.adjust	geneID	Count
R-HSA-8939242	RUNX1 regulates transcription of genes involved in differentiation of keratinocytes	8/10619	0.026027071	*RUNX1/SOCS3*	2
R-HSA-909733	Interferon alpha/beta signaling	69/10619	0.047776744	*IFIT3/XAF1/SOCS3*	3
R-HSA-449147	Signaling by Interleukins	463/10619	0.047776744	*IL1RL1/SERPINB2/ANXA1/CSF1/ALOX15/SOCS3*	6
R-HSA-2142691	Synthesis of Leukotrienes (LT) and Eoxins (EX)	21/10619	0.047776744	*ALOX15/GGT5*	2
R-HSA-6785807	Interleukin-4 and Interleukin-13 signaling	108/10619	0.066692828	*ANXA1/ALOX15/SOCS3*	3
R-HSA-9025106	Biosynthesis of DPAn-6 SPMs	2/10619	0.066692828	*ALOX15*	1
R-HSA-9026286	Biosynthesis of DPAn-3-derived protectins and resolvins	2/10619	0.066692828	*ALOX15*	1
R-HSA-8948216	Collagen chain trimerization	44/10619	0.066692828	*COL15A1/COL11A2*	2
R-HSA-5660489	MTF1 activates gene expression	3/10619	0.066692828	*CSRP1*	1
R-HSA-9014843	Interleukin-33 signaling	3/10619	0.066692828	*IL1RL1*	1
R-HSA-8848021	Signaling by PTK6	54/10619	0.066692828	*EPAS1/SOCS3*	2
R-HSA-9006927	Signaling by Non-Receptor Tyrosine Kinases	54/10619	0.066692828	*EPAS1/SOCS3*	2
R-HSA-2142753	Arachidonic acid metabolism	59/10619	0.066692828	*ALOX15/GGT5*	2
R-HSA-8849474	PTK6 Activates STAT3	4/10619	0.066692828	*SOCS3*	1
R-HSA-8941333	RUNX2 regulates genes involved in differentiation of myeloid cells	4/10619	0.066692828	*RUNX1*	1
R-HSA-9018681	Biosynthesis of protectins	4/10619	0.066692828	*ALOX15*	1
R-HSA-9018683	Biosynthesis of DPA-derived SPMs	4/10619	0.066692828	*ALOX15*	1

R-HSA-9023661	Biosynthesis of E-series 18(R)-resolvins	4/10619	0.066692828	*ALOX15*	1
R-HSA-9025094	Biosynthesis of DPAn-3 SPMs	4/10619	0.066692828	*ALOX15*	1
R-HSA-2022090	Assembly of collagen fibrils and other multimeric structures	61/10619	0.066692828	*COL15A1/COL11A2*	2
R-HSA-1442490	Collagen degradation	64/10619	0.066692828	*COL15A1/COL11A2*	2
R-HSA-391906	Leukotriene receptors	5/10619	0.066692828	*CYSLTR2*	1
R-HSA-8849473	PTK6 Expression	5/10619	0.066692828	*EPAS1*	1
R-HSA-8935964	RUNX1 regulates expression of components of tight junctions	5/10619	0.066692828	*RUNX1*	1
R-HSA-8939247	RUNX1 regulates transcription of genes involved in interleukin signaling	5/10619	0.066692828	*RUNX1*	1
R-HSA-9018896	Biosynthesis of E-series 18(S)-resolvins	5/10619	0.066692828	*ALOX15*	1
R-HSA-1650814	Collagen biosynthesis and modifying enzymes	67/10619	0.066692828	*COL15A1/COL11A2*	2
R-HSA-913531	Interferon Signaling	199/10619	0.066692828	*IFIT3/XAF1/SOCS3*	3
R-HSA-2142770	Synthesis of 15-eicosatetraenoic acid derivatives	6/10619	0.066692828	*ALOX15*	1
R-HSA-8931987	RUNX1 regulates estrogen receptor mediated transcription	6/10619	0.066692828	*RUNX1*	1
R-HSA-8939245	RUNX1 regulates transcription of genes involved in BCR signaling	6/10619	0.066692828	*RUNX1*	1
R-HSA-8939256	RUNX1 regulates transcription of genes involved in WNT signaling	6/10619	0.066692828	*RUNX1*	1
R-HSA-9018679	Biosynthesis of EPA-derived SPMs	6/10619	0.066692828	*ALOX15*	1

R-HSA-2142712	Synthesis of 12-eicosatetraenoic acid derivatives	7/10619	0.073269005	*ALOX15*	1
R-HSA-8939246	RUNX1 regulates transcription of genes involved in differentiation of myeloid cells	7/10619	0.073269005	*RUNX1*	1
R-HSA-416476	G alpha (q) signalling events	217/10619	0.073611597	*RGS1/ANXA1/CYSLTR2*	3
R-HSA-444473	Formyl peptide receptors bind formyl peptides and many other ligands	8/10619	0.079109245	*ANXA1*	1
R-HSA-1474290	Collagen formation	90/10619	0.084326826	*COL15A1/COL11A2*	2
R-HSA-8951936	RUNX3 regulates p14-ARF	9/10619	0.084326826	*RUNX1*	1
R-HSA-549127	Organic cation transport	10/10619	0.089012925	*RUNX1*	1
R-HSA-8877330	RUNX1 and FOXP3 control the development of regulatory T lymphocytes (Tregs)	10/10619	0.089012925	*RUNX1*	1
R-HSA-1059683	Interleukin-6 signaling	11/10619	0.091122623	*SOCS3*	1
R-HSA-1234158	Regulation of gene expression by Hypoxia-inducible Factor	11/10619	0.091122623	*EPAS1*	1
R-HSA-2586552	Signaling by Leptin	11/10619	0.091122623	*SOCS3*	1
R-HSA-174403	Glutathione synthesis and recycling	12/10619	0.097074333	*GGT5*	1

Supplemental Table 2: Gene Ontology Terms Upregulated in *JAK1*^{GOF} human whole blood and iPSC

GO Term	Description	P-value	FDR q-value	Enrichment	N	B	n	b	Genes
GO:0061737	leukotriene signaling pathway	4.27E-05	6.71E-01	190.87	19564	5	41	2	[*CYSLTR2* - cysteinyl leukotriene receptor 2, *RGS1* - regulator of g-protein signaling 1]
GO:0002683	negative regulation of immune system process	5.89E-05	4.63E-01	6.9	19564	484	41	7	[*SAMSN1* - sam domain, sh3 domain and nuclear localization signals 1, *ALOX15* - arachidonate 15-lipoxygenase, *TMEM176B* - transmembrane protein 176b, *TMEM176A* - transmembrane protein 176a, *ANXA1* - annexin a1, *IL1RL1* - interleukin 1 receptor-like 1, *RUNX1* - runt-related transcription factor 1]
GO:2001199	negative regulation of dendritic cell differentiation	6.39E-05	3.35E-01	159.06	19564	6	41	2	[*TMEM176B* - transmembrane protein 176b, *TMEM176A* - transmembrane protein 176a]
GO:0019221	cytokine-mediated signaling pathway	6.51E-05	2.56E-01	5.68	19564	672	41	8	[*XAF1* - xiap associated factor 1, *CSF1* - colony stimulating factor 1 (macrophage), *IFIT3* - interferon-induced protein with tetratricopeptide repeats 3, *ALOX15* - arachidonate 15-lipoxygenase, *SOCS3* - suppressor of cytokine signaling 3, *ANXA1* - annexin a1, *SERPINB2* - serpin peptidase inhibitor, clade b (ovalbumin), member 2, *IL1RL1* - interleukin 1 receptor-like 1]
GO:1902106	negative regulation of leukocyte differentiation	6.56E-05	2.06E-01	18.35	19564	104	41	4	[*TMEM176B* - transmembrane protein 176b, *ANXA1* - annexin a1, *TMEM176A* - transmembrane protein 176a, *RUNX1* - runt-related transcription factor 1]
GO:0045595	regulation of cell differentiation	8.86E-05	2.32E-01	3.25	19564	1907	41	13	[*CSF1* - colony stimulating factor 1 (macrophage), *BHLHE40* - basic helix-loop-helix family, member e40, *HOXB4* - homeobox b4, *SOCS3* - suppressor of cytokine signaling 3, *TMEM176B* - transmembrane protein 176b, *GRHL1* - grainyhead-like 1 (drosophila), *RUNX1* - runt-related transcription factor 1, *KIAA1024* - kiaa1024, *ANXA1* - annexin a1, *TMEM176A* - transmembrane protein 176a, *WNT11* - wingless-type mmtv integration site family, member 11,

									PKP2 - plakophilin 2, *PMP22* - peripheral myelin protein 22]
GO:0002820	negative regulation of adaptive immune response	1.22E-04	2.73E-01	31.12	19564	46	41	3	[*SAMSN1* - sam domain, sh3 domain and nuclear localization signals 1, *ALOX15* - arachidonate 15-lipoxygenase, *IL1RL1* - interleukin 1 receptor-like 1]
GO:0034097	response to cytokine	1.78E-04	3.49E-01	5.78	19564	578	41	7	[*XAF1* - xiap associated factor 1, *IFIT3* - interferon-induced protein with tetratricopeptide repeats 3, *ALOX15* - arachidonate 15-lipoxygenase, *SOCS3* - suppressor of cytokine signaling 3, *LAMP3* - lysosomal-associated membrane protein 3, *ANXA1* - annexin a1, *GGT5* - gamma-glutamyltransferase 5]
GO:0002934	desmosome organization	1.91E-04	3.33E-01	95.43	19564	10	41	2	[*PKP2* - plakophilin 2, *GRHL1* - grainyhead-like 1 (drosophila)]
GO:1902105	regulation of leukocyte differentiation	3.04E-04	4.78E-01	8.4	19564	284	41	5	[*CSF1* - colony stimulating factor 1 (macrophage), *TMEM176B* - transmembrane protein 176b, *TMEM176A* - transmembrane protein 176a, *ANXA1* - annexin a1, *RUNX1* - runt-related transcription factor 1]
GO:0050777	negative regulation of immune response	3.21E-04	4.58E-01	12.16	19564	157	41	4	[*ALOX15* - arachidonate 15-lipoxygenase, *SAMSN1* - sam domain, sh3 domain and nuclear localization signals 1, *ANXA1* - annexin a1, *IL1RL1* - interleukin 1 receptor-like 1]
GO:1903707	negative regulation of hemopoiesis	3.21E-04	4.20E-01	12.16	19564	157	41	4	[*TMEM176B* - transmembrane protein 176b, *ANXA1* - annexin a1, *TMEM176A* - transmembrane protein 176a, *RUNX1* - runt-related transcription factor 1]
GO:2001198	regulation of dendritic cell differentiation	3.29E-04	3.98E-01	73.41	19564	13	41	2	[*TMEM176B* - transmembrane protein 176b, *TMEM176A* - transmembrane protein 176a]
GO:0002819	regulation of adaptive immune response	4.05E-04	4.55E-01	11.43	19564	167	41	4	[*ALOX15* - arachidonate 15-lipoxygenase, *SAMSN1* - sam domain, sh3 domain and nuclear localization signals 1, *ANXA1* - annexin a1, *IL1RL1* - interleukin 1 receptor-like 1]
GO:0051239	regulation of multicellular organismal process	6.99E-04	7.32E-01	2.28	19564	3342	41	16	[*CSF1* - colony stimulating factor 1 (macrophage), *BHLHE40* - basic helix-loop-helix family, member e40, *TMEM176B* - transmembrane protein 176b, *GADD45G* - growth arrest and dna-damage-inducible,

									gamma, *IL1RL1* - interleukin 1 receptor-like 1, *GRHL1* - grainyhead-like 1 (drosophila), *RUNX1* - runt-related transcription factor 1, *EPAS1* - endothelial pas domain protein 1, *PDK4* - pyruvate dehydrogenase kinase, isozyme 4, *KIAA1024* - kiaa1024, *WNT11* - wingless-type mmtv integration site family, member 11, *ANXA1* - annexin a1, *TMEM176A* - transmembrane protein 176a, *SERPINB2* - serpin peptidase inhibitor, clade b (ovalbumin), member 2, *PKP2* - plakophilin 2, *PMP22* - peripheral myelin protein 22]
GO:0048856	anatomical structure development	7.73E-04	7.59E-01	2.26	19564	3372	41	16	[*CSF1* - colony stimulating factor 1 (macrophage), *ALOX15* - arachidonate 15-lipoxygenase, *HOXB4* - homeobox b4, *SOCS3* - suppressor of cytokine signaling 3, *GADD45G* - growth arrest and dna-damage-inducible, gamma, GRHL1 - grainyhead-like 1 (drosophila), *RUNX1* - runt-related transcription factor 1, *EPAS1* - endothelial pas domain protein 1, *WNT11* - wingless-type mmtv integration site family, member 11, *ANXA1* - annexin a1, *PKP2* - plakophilin 2, *COL11A2* - collagen, type xi, alpha 2, *CSRP1* - cysteine and glycine-rich protein 1, *PMP22* - peripheral myelin protein 22, *RHOBTB3* - rho-related btb domain containing 3, *HIC1* - hypermethylated in cancer 1]
GO:0043371	negative regulation of CD4-positive, alpha-beta T cell differentiation	7.95E-04	7.35E-01	47.72	19564	20	41	2	[*ANXA1* - annexin a1, *RUNX1* - runt-related transcription factor 1]
GO:0045596	negative regulation of cell differentiation	8.73E-04	7.62E-01	4.44	19564	753	41	7	[*TMEM176B* - transmembrane protein 176b, *KIAA1024* - kiaa1024, TMEM176A - transmembrane protein 176a, *ANXA1* - annexin a1, *PKP2* - plakophilin 2, *PMP22* - peripheral myelin protein 22, *RUNX1* - runt-related transcription factor 1]
GO:0035455	response to interferon-alpha	8.77E-04	7.26E-01	45.44	19564	21	41	2	*[IFIT3* - interferon-induced protein with tetratricopeptide repeats 3, *LAMP3* - lysosomal-associated membrane protein 3]

GO:0050793	regulation of developmental process	9.56E-04	7.51E-01	2.43	19564	2750	41	14	[*CSF1* - colony stimulating factor 1 (macrophage), *BHLHE40* - basic helix-loop-helix family, member e40, *HOXB4* - homeobox b4, TMEM176B - transmembrane protein 176b, *SOCS3* - suppressor of cytokine signaling 3, *GRHL1* - grainyhead-like 1 (drosophila), *RUNX1* - runt-related transcription factor 1, *KIAA1024* - kiaa1024, *ANXA1* - annexin a1, *TMEM176A* - transmembrane protein 176a, *WNT11* - wingless-type mmtv integration site family, member 11, *PKP2* - plakophilin 2, *PMP22* - peripheral myelin protein 22, *RHOBTB3* - rho-related btb domain containing 3]

Supplemental Table 3: Upregulated cytokines and cytokine receptors in *JAK1*^{GOF} iPSC

Gene	logFC	logCPM	LR	PValue	FDR	Fold_change
TNFSF15	3.797622663	1.574209313	191.9122669	1.22E-43	5.21E-41	13.90587541
IL1RL1	3.700639903	-1.002211755	30.14791207	4.00E-08	5.03E-07	13.00180397
CCL25	3.498303831	-2.074241697	14.17174094	0.000166858	0.00092517	11.30041485
TNFSF8	3.303979269	2.05767837	153.024199	3.78E-35	9.97E-33	9.876358936
HGF	2.597267721	2.189966103	59.60791743	1.16E-14	4.18E-13	6.051394845
AMH	2.327224062	-0.089148548	41.81089916	1.01E-10	1.94E-09	5.018388152
ACVRL1	2.205370869	2.743793532	33.74419083	6.29E-09	9.16E-08	4.611930809
IL1R1	2.157625983	3.464173337	120.3960164	5.18E-28	7.93E-26	4.461800423
CXCL14	2.156592606	4.405573365	75.34488637	3.95E-18	2.21E-16	4.458605659
IL11	2.119332922	1.011441443	32.76362551	1.04E-08	1.45E-07	4.344929963
EDAR	2.083085156	1.353273714	59.75340629	1.08E-14	3.92E-13	4.23712343
IL6ST	1.854464862	7.176923736	38.86797289	4.53E-10	7.90E-09	3.616175915
TSLP	1.776622428	0.474724994	15.78169038	7.11E-05	0.00043732	3.426231012
IL6	1.721409529	-1.074578625	12.82294766	0.000342394	0.001726759	3.297584272
CXCL16	1.538196414	-1.073105573	8.540244691	0.003473789	0.012727864	2.904311937
MET	1.522841419	3.99730997	97.63306945	5.04E-23	4.77E-21	2.873564473
TGFB3	1.433450483	0.911489221	10.55602672	0.001158099	0.00499917	2.700919203
GHR	1.40935535	1.683837483	25.63010331	4.14E-07	4.24E-06	2.65618448
CSF1	1.206346229	3.980694392	92.07395842	8.35E-22	7.01E-20	2.307524921
PDGFB	1.092265312	1.906287327	29.33745258	6.08E-08	7.40E-07	2.132085527

Supplemental Table 4: Upregulated cytokines and cytokine receptors in *JAK1*^{GOF} whole blood

Gene	logFC	logCPM	LR	PValue	FDR	Fold_change
CCL23	5.532586773	4.300968532	68.81635467	1.08E-16	2.84E-14	46.28865613
IL1RL1	4.701646072	3.44894793	126.8499243	2.00E-29	1.88E-26	26.02174979
IL5RA	3.130320452	5.632036485	155.4982861	1.09E-35	1.81E-32	8.756294348
IL2RA	2.583645639	4.67347723	76.25282684	2.50E-18	8.50E-16	5.994525824
IL17RB	2.506346253	2.507024878	35.66977875	2.34E-09	1.84E-07	5.681792926
CSF1	2.374445474	5.569597882	84.41210077	4.02E-20	1.67E-17	5.185364745
CCR3	1.973900741	6.816148993	48.2575015	3.74E-12	5.64E-10	3.928288103
OSM	1.870495465	4.115602021	9.829304852	0.001717532	0.019952587	3.656581366
VEGFA	1.707410571	3.958312041	40.56935023	1.90E-10	1.85E-08	3.265741437
ACVR1B	1.131870582	4.843748804	14.7223467	0.000124561	0.002557515	2.19142694
CLCF1	1.11809965	2.685192364	12.5492282	0.00039637	0.006502986	2.170608667

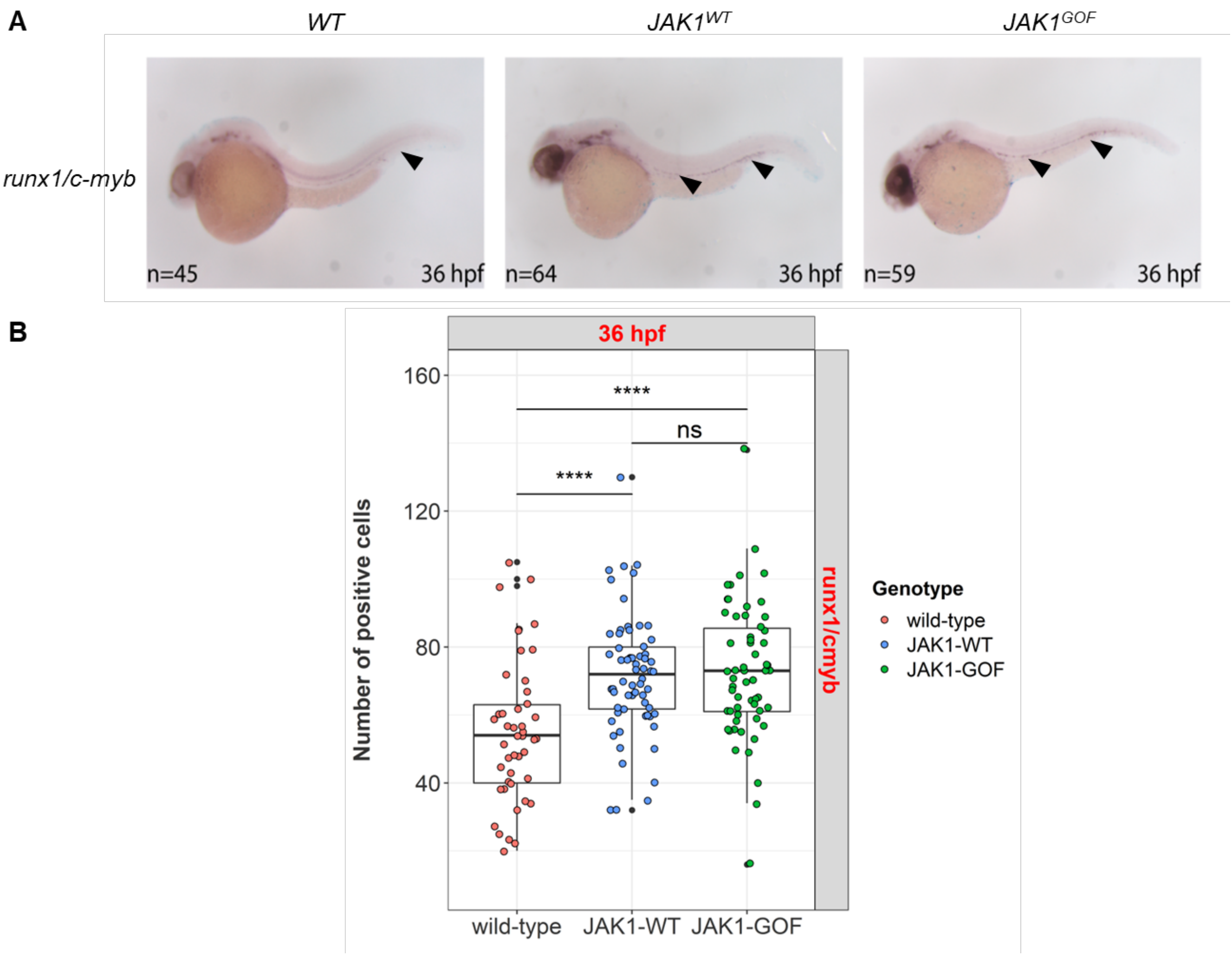

Supplemental figure 4: *JAK1*^{GOF} transgenic zebrafish have higher levels of hematopoietic stem cells (HPSCs). A) WISH using digoxigenin-labeled RNA antisense probes for *runx1/c-myb* at 36 hpf in wild-type (WT), *JAK1*^{WT} and *JAK1*^{GOF} transgenics. A representative micrograph of each genotype and time point is shown. B) Plots of runx1/c-myb -positive cell counts for each genotype. Each individual embryo count is indicated by a filled circle and the boxplot shows quartile distribution with whiskers covering 95% confidence interval. One-way analysis of variance was used to quantify the statistical differences between the groups. Legend: ns - $p > 0.05$; **** - $p \leq 0.0001$.

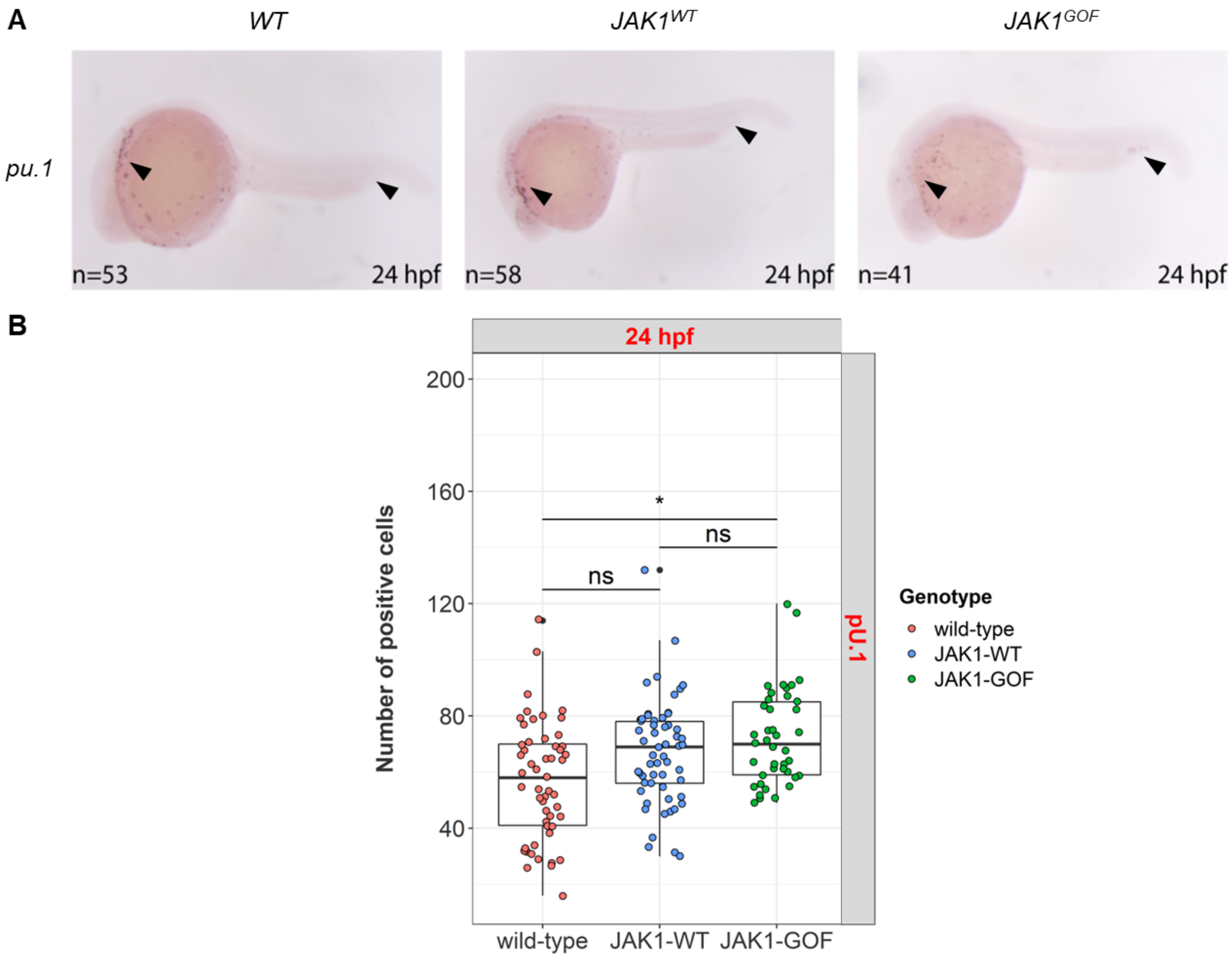

Supplemental figure 5: *JAK1*^{GOF} transgenic zebrafish have higher levels of early myeloid progenitors during hematopoiesis in zebrafish. A) WISH using a digoxigenin-labeled RNA antisense probe for *pu.1* at 24 hpf in wild-type (WT), *JAK1*^{WT} and *JAK1*^{GOF} transgenics. A representative micrograph of each genotype is shown. B) Plots of *pu.1*-positive cell counts for each genotype. Each individual embryo count is indicated by a filled circle and the boxplot shows quartile distribution with whiskers covering 95% confidence interval. One-way analysis of variance was used to quantify the statistical differences between the groups. Legend: ns - $p > 0.05$; * - $p \leq 0.05$.

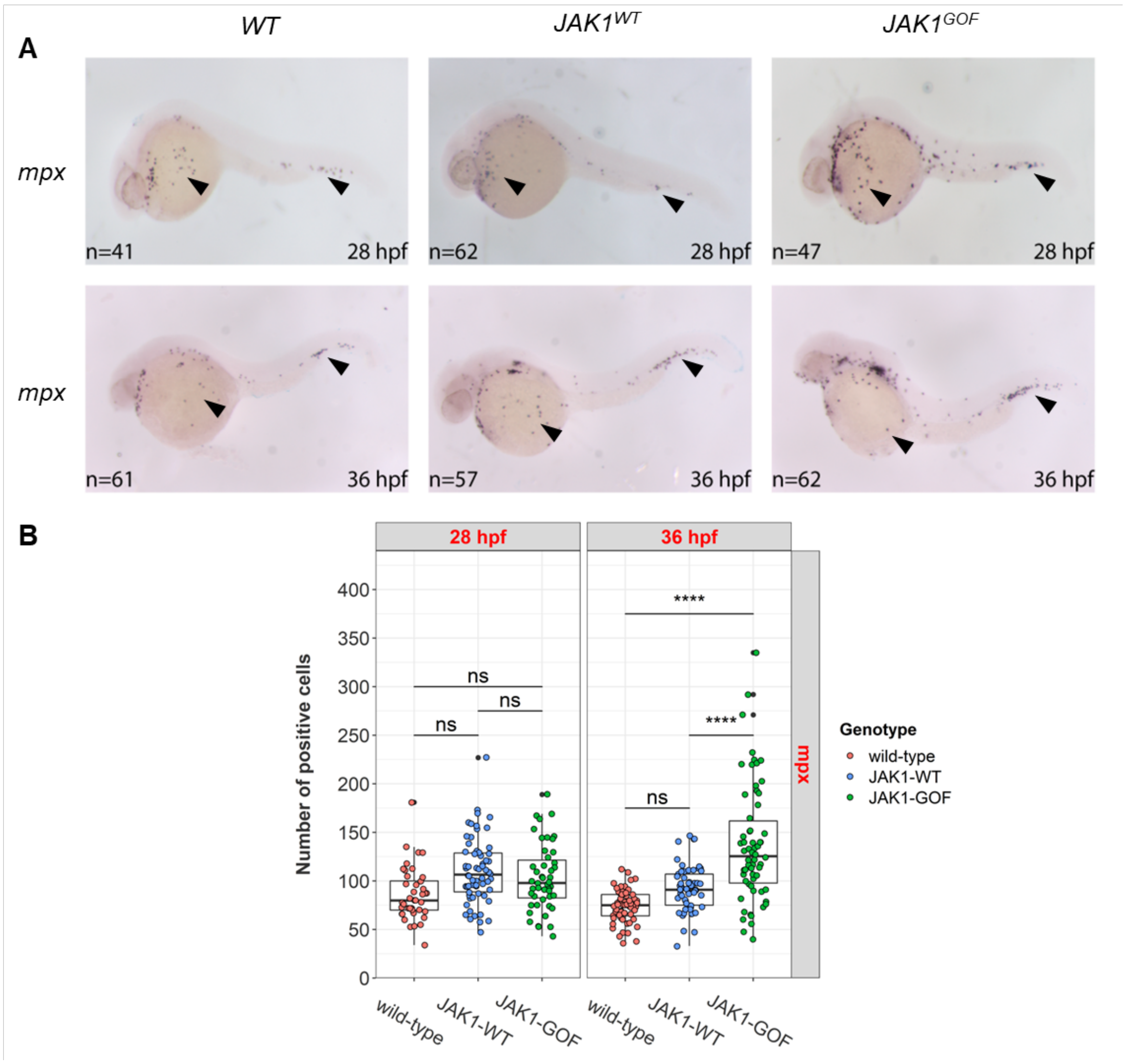

Supplemental figure 6: *JAK1*^{GOF} transgenic zebrafish have higher levels of neutrophils during hematopoiesis in zebrafish. A) WISH using a digoxigenin-labeled RNA antisense probe for *mpx* at 28 and 36 hpf in wild-type (WT), *JAK1*^{WT} and *JAK1*^{GOF} transgenics. A representative micrograph of each genotype and time point is shown. B) Plots of *mpx*-positive cell counts for each genotype and time point. Each individual embryo count is indicated by a filled circle and the boxplot shows quartile distribution with whiskers covering 95% confidence interval. One-way analysis of variance was used to quantify the statistical differences between the groups. Legend: ns - $p > 0.05$; **** - $p \leq 0.0001$.

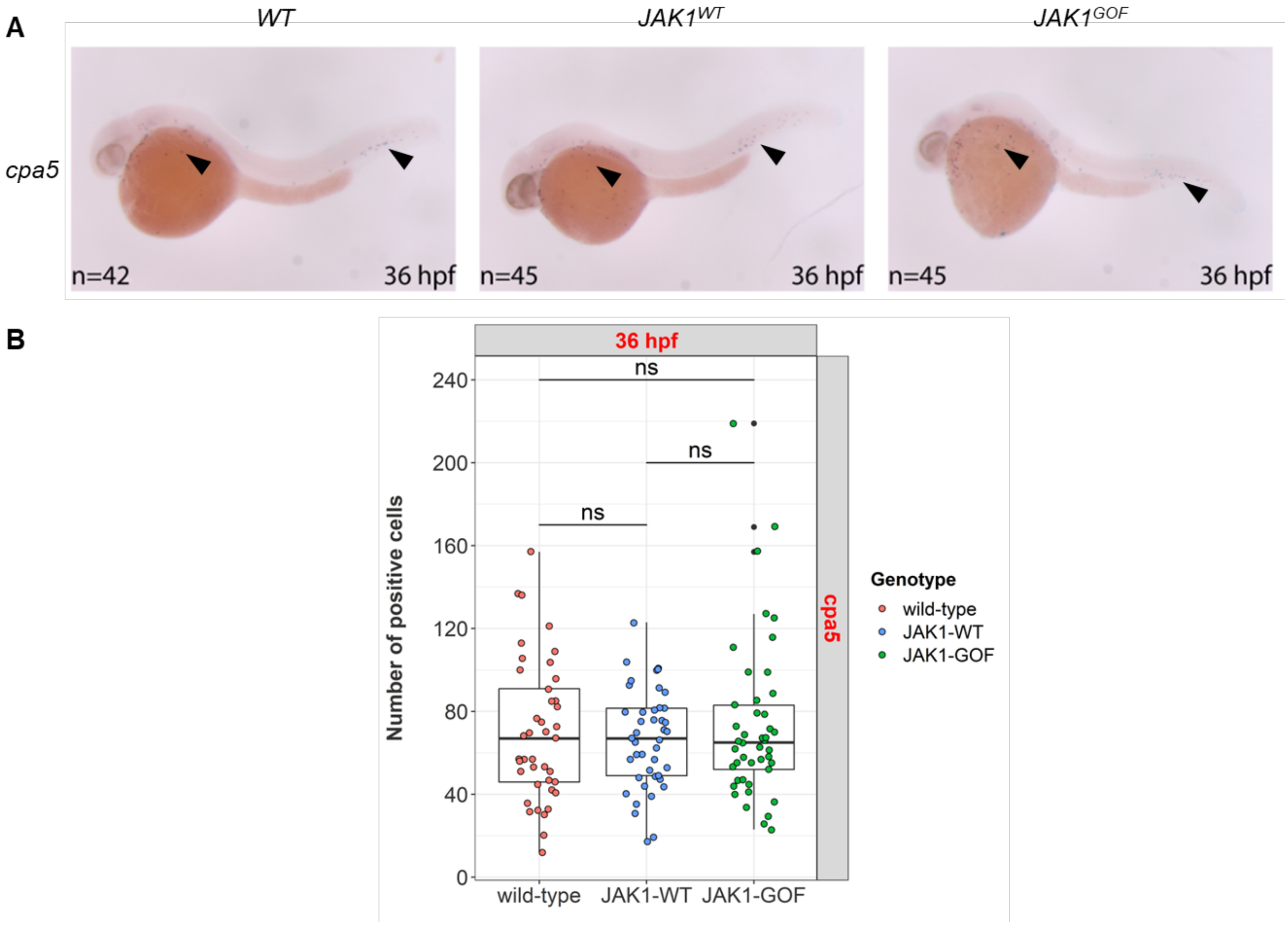

Supplemental figure 7: *JAK1*^{GOF} transgenic zebrafish have comparable levels of mast cells to controls at 36 hpf in zebrafish. A) WISH using a digoxigenin-labeled RNA antisense probe for *cpa5* at 36 hpf in wild-type (WT), *JAK1*^{WT} and *JAK1*^{GOF} transgenics. A representative micrography of each genotype and time point is shown. B) Plots of *cpa5*-positive cell counts for each genotype. Each individual embryo count is indicated by a filled circle and the boxplot shows quartile distribution with whiskers covering 95% confidence interval. One-way analysis of variance was used to quantify the statistical differences between the groups. Legend: ns - $p > 0.05$.

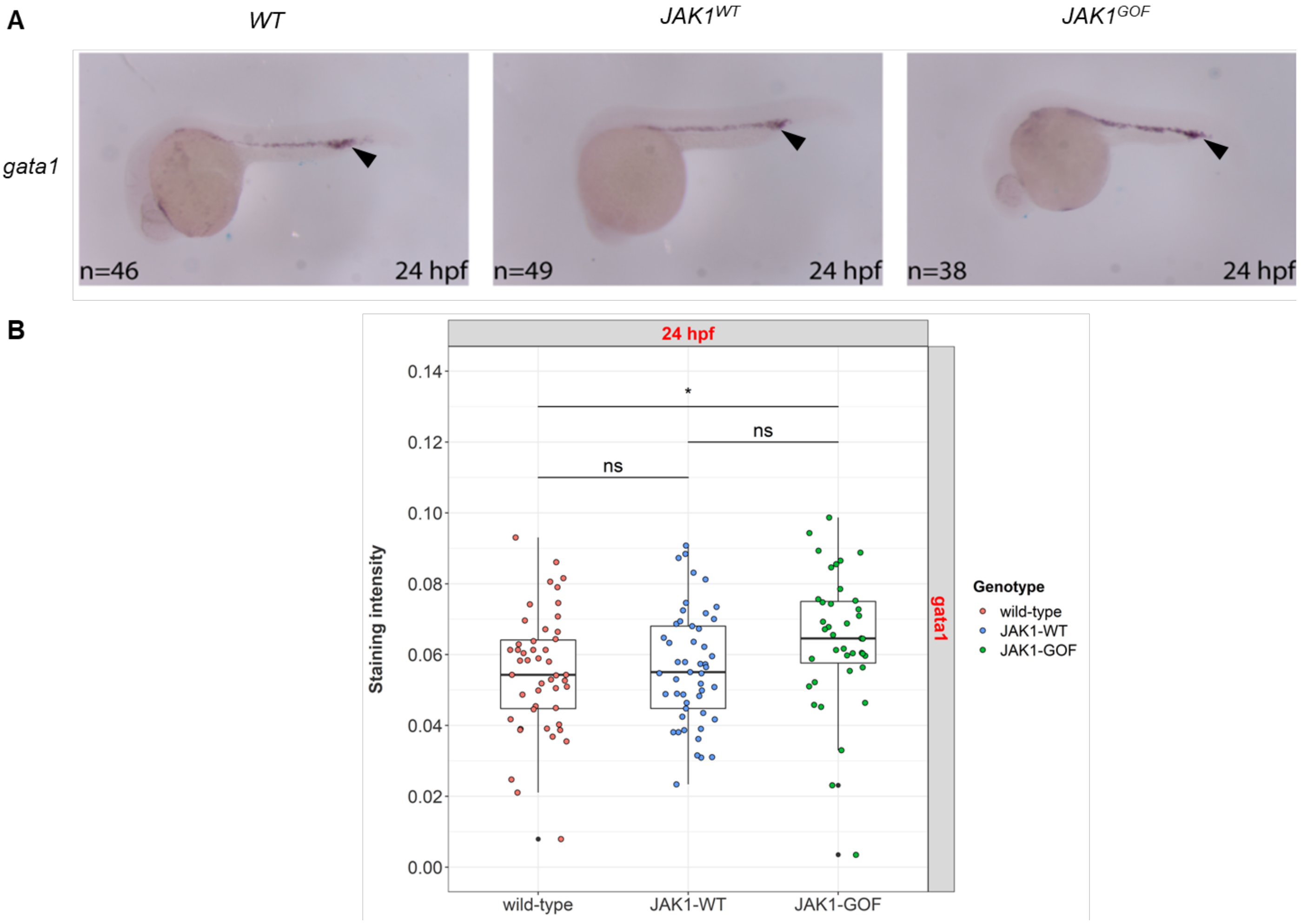

Supplemental figure 8: *JAK1*^{GOF} transgene impacts red blood cell hematopoiesis in zebrafish.

A) WISH using a digoxigenin-labeled RNA antisense probe targeting *gata1* at 24 hpf. B) Plots of *gata1* staining quantification for each genotype at 24hpf in wild-type (WT), *JAK1*^{WT} and *JAK1*^{GOF} transgenics. Each filled circle represents the staining intensity for each embryo and the boxplot shows quartile distribution with whiskers covering 95% confidence interval. One-way analysis of variance was used to quantify the statistical differences between the groups. Legend: ns - $p > 0.05$; * - $p < 0.05$.

S9A

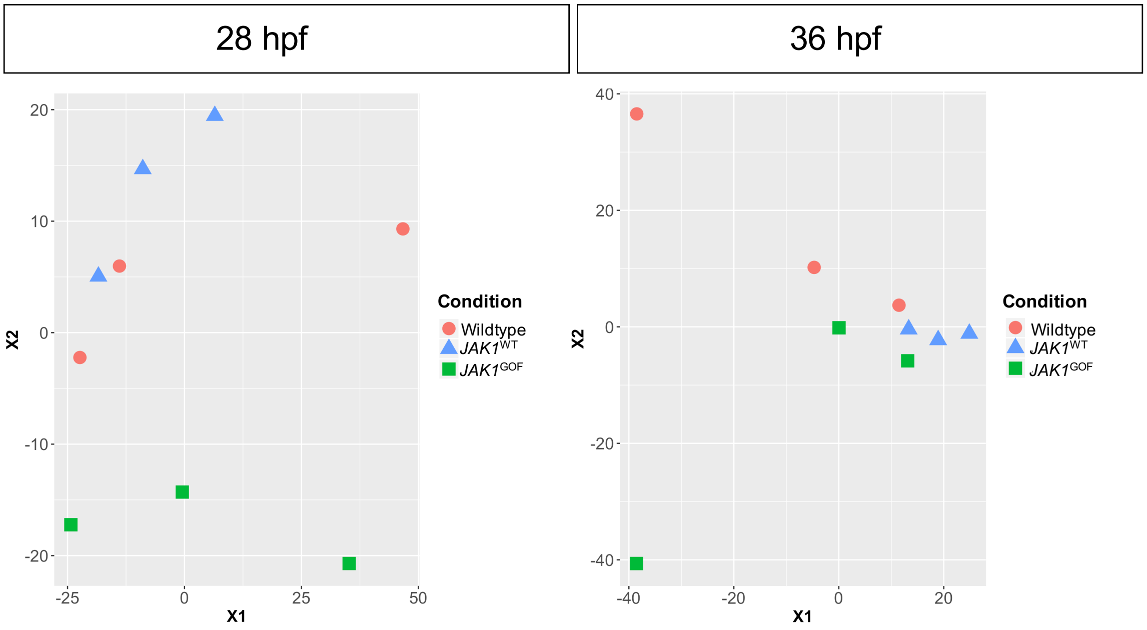

S9B

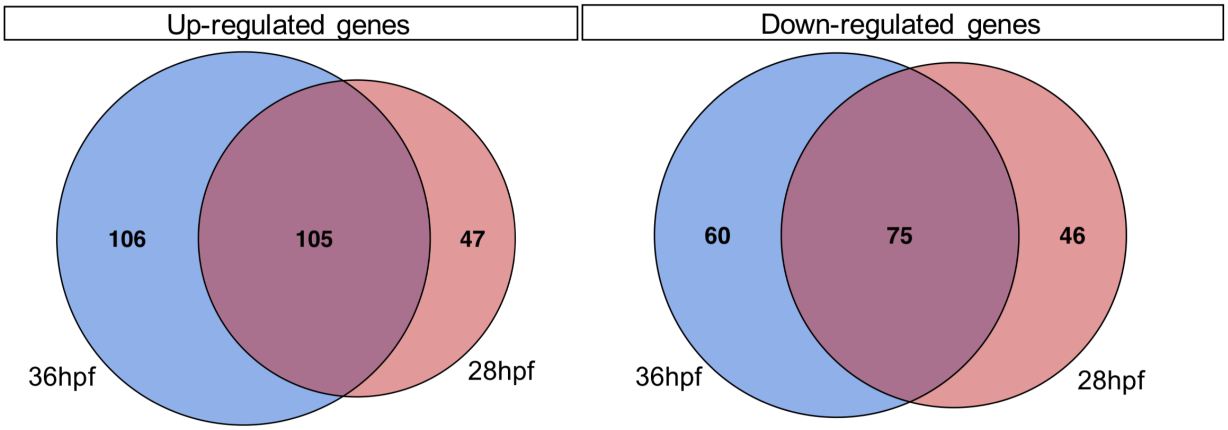

Supplemental figure 9: A) Zebrafish gene expression analysis. Multidimensional Scaling (MDS) of wildtype, *JAK1*^{GOF} and *JAK1*^{WT} transgenic zebrafish. The first 2 components (X1 and X2) were plotted. B) Venn diagrammatic representation of up-regulated and down-regulated genes at 28 and 36 hpf.

S10A

Sample	Sex	Status
T_Pt1_c16_p14	Male	Normal (Sex Difference)
T_Pt1_c15_p14	Male	Normal (Sex Difference)

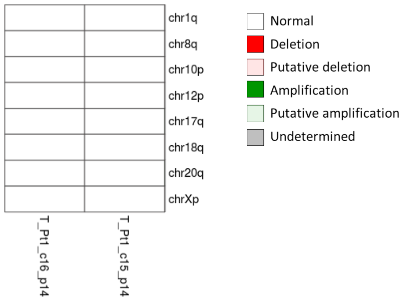

S10B

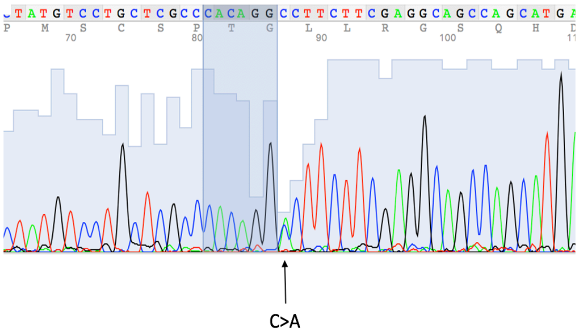

S10C

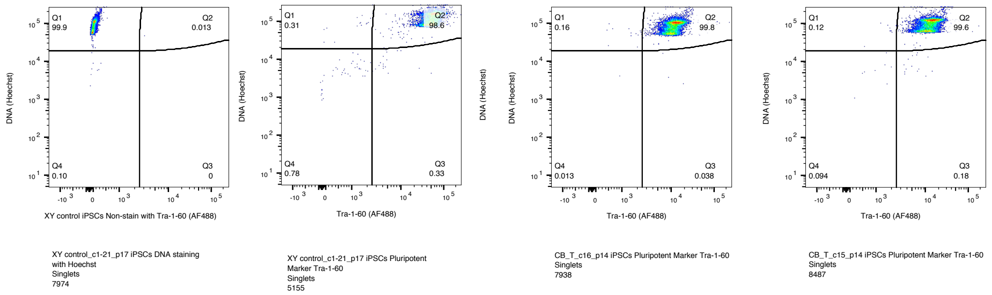

Supplemental figure 10: iPSC line generation: A) Normal karyotype from *JAK1*^{GOF} iPSC. B) PCR analysis from *JAK1*^{GOF} iPSC line confirms that it carries the desired c.1901 C>A pathogenic variant in *JAK1*. C) Flow cytometry demonstrating pluripotency as indicated by increased Tra-1-60 expression in XY control and *JAK1*^{GOF} iPSC lines.

Supplemental Table 5: Gene ontology term analysis of genes upregulated in *JAK1*^{GOF} zebrafish compared to *JAK1*^{WT} at both 28 and 36hpf

GO Term	Description	P-value	FDR q-value	Enrichment	N	B	n	b	Genes
GO:0009615	response to virus	6.94E-08	5.64E-04	27.38	12298	35	77	6	[*irf7* - interferon regulatory factor 7, *mxb* - myxovirus (influenza) resistance b, *rsad2* - radical s-adenosyl methionine domain containing 2, *tmem173* - transmembrane protein 173, *irf1b* - interferon regulatory factor 1b, *mxa* - myxovirus (influenza) resistance a]
GO:0043207	response to external biotic stimulus	2.64E-07	1.07E-03	9.98	12298	144	77	9	[*irf7* - interferon regulatory factor 7, *irg1l* - immunoresponsive gene 1, like, *mxb* - myxovirus (influenza) resistance b, *cyp51* - cytochrome p450, family 51, *junba* - jun b proto-oncogene a, *rsad2* - radical s-adenosyl methionine domain containing 2, *tmem173* - transmembrane protein 173, *irf1b* - interferon regulatory factor 1b, *mxa* - myxovirus (influenza) resistance a]
GO:0009607	response to biotic stimulus	2.97E-07	8.04E-04	9.85	12298	146	77	9	[*irf7* - interferon regulatory factor 7, *irg1*l - immunoresponsive gene 1, like, *mxb* - myxovirus (influenza) resistance b, *cyp51* - cytochrome p450, family 51, *junba* - jun b proto-oncogene a, *rsad2* - radical s-adenosyl methionine domain containing 2, *tmem173* - transmembrane protein 173, *irf1b* - interferon regulatory factor 1b, *mxa* - myxovirus (influenza) resistance a]
GO:0051707	response to other organism	7.31E-07	1.48E-03	10.74	12298	119	77	8	[*irf7* - interferon regulatory factor 7, *irg1l* - immunoresponsive gene 1, like, *mxb* - myxovirus (influenza) resistance b, *cyp51* - cytochrome p450, family 51, *rsad2* - radical s-adenosyl methionine domain containing 2, *tmem173* - transmembrane protein 173, *irf1b* - interferon regulatory factor 1b, *mxa* - myxovirus (influenza) resistance a]
GO:0051704	multi-organism process	1.35E-06	2.20E-03	9.9	12298	129	77	8	[*irf7* - interferon regulatory factor 7, *irg1l* - immunoresponsive gene 1, like, *mxb* - myxovirus (influenza) resistance b, *cyp51* - cytochrome

									p450, family 51, *rsad2* - radical s-adenosyl methionine domain containing 2, *tmem173* - transmembrane protein 173, *irf1b* - interferon regulatory factor 1b, *mxa* - myxovirus (influenza) resistance a]
GO:0015671	oxygen transport	6.45E-05	8.73E-02	36.86	12298	13	77	3	[*hbae1* - hemoglobin alpha embryonic-1, *hbbe2* - hemoglobin beta embryonic-2, *hbbe1.1* - hemoglobin beta embryonic-1.1]
GO:0009605	response to external stimulus	9.22E-05	1.07E-01	4.86	12298	296	77	9	[*irf7* - interferon regulatory factor 7, *irg1l* - immunoresponsive gene 1, like, *mxb* - myxovirus (influenza) resistance b, *cyp51* - cytochrome p450, family 51*, junba* - jun b proto-oncogene a, *rsad2* - radical s-adenosyl methionine domain containing 2, *tmem173* - transmembrane protein 173, *irf1b* - interferon regulatory factor 1b, *mxa* - myxovirus (influenza) resistance a]
GO:0015669	gas transport	1.25E-04	1.26E-01	29.95	12298	16	77	3	[*hbae1* - hemoglobin alpha embryonic-1, *hbbe1.1* - hemoglobin beta embryonic-1.1, *hbbe2* - hemoglobin beta embryonic-2]
GO:0050776	regulation of immune response	2.32E-04	2.10E-01	8.97	12298	89	77	5	[*socs1a* - suppressor of cytokine signaling 1a, *b2m* - beta-2-microglobulin, *zgc:174863* - zgc:174863, *tmem173* - transmembrane protein 173, *irf1b* - interferon regulatory factor 1b]
GO:0002376	immune system process	3.61E-04	2.93E-01	4.55	12298	281	77	8	[*irf3* - interferon regulatory factor 3, *irf7* - interferon regulatory factor 7, *b2m* - beta-2-microglobulin, *rsad2* - radical s-adenosyl methionine domain containing 2, *zgc:174863* - zgc:174863, *tmem173* - transmembrane protein 173, *mhc1zba* - major histocompatibility complex class i zba, *irf1b* - interferon regulatory factor 1b]
GO:0045824	negative regulation of innate immune response	5.71E-04	4.22E-01	53.24	12298	6	77	2	[*socs1a* - suppressor of cytokine signaling 1a, *b2m* - beta-2-microglobulin]
GO:0002682	regulation of immune system process	7.60E-04	5.14E-01	5.51	12298	174	77	6	*[irf7* - interferon regulatory factor 7, *socs1a* - suppressor of cytokine signaling 1a, *b2m* - beta-2-microglobulin, *zgc:174863* - zgc:174863, *tmem173* -

									transmembrane protein 173, *irf1b* - interferon regulatory factor 1b]
GO:0050688	regulation of defense response to virus	7.96E-04	4.97E-01	45.63	12298	7	77	2	[*tmem173* - transmembrane protein 173, *irf1b* - interferon regulatory factor 1b]
GO:0031347	regulation of defense response	8.08E-04	4.68E-01	9.54	12298	67	77	4	[*socs1a* - suppressor of cytokine signaling 1a, *b2m* - beta-2-microglobulin, *tmem173* - transmembrane protein 173, *irf1b* - interferon regulatory factor 1b]
